# Supplementary material for: Complex skin modes in non-Hermitian coupled laser arrays
Source: Light Sci Appl. 2022 Nov 28;11:336. doi: 10.1038/s41377-022-01030-0 (PMC9705320; doi:10.1038/s41377-022-01030-0)
Supplement: Supplementary file 1 — Supplementary Information [file 41377_2022_1030_MOESM1_ESM.docx]

Supplementary Information for

**Complex Skin Modes in Non-Hermitian Coupled Laser Arrays**

Yuzhou G. N. Liu^1^, Yunxuan Wei^1^, Omid Hemmatyar^1^, Georgios G. Pyrialakos^2^,

Pawel S. Jung^2,3^, Demetrios N. Christodoulides^2^, Mercedeh Khajavikhan^1,4,*^

^1^Ming Hsieh Department of Electrical and Computer Engineering, University of Southern California, Los Angeles, California 90089, USA

^2^CREOL, The College of Optics & Photonics, University of Central Florida, Orlando, Florida 32816–2700, USA

^3^Faculty of Physics, Warsaw University of Technology, Koszykowa 75, 00-662 Warsaw, Poland

^4^Department of Physics & Astronomy, Dornsife College of Letters, Arts, & Sciences, University of Southern California, Los Angeles, California 90089, USA

*Corresponding author: [khajavik@usc.edu](mailto:khajavik@usc.edu)

**Section 1. Coupled mode theory analysis for feedback-promoted unidirectional lasing in microring lasers**

To analyze the mode direction in the ring resonator, we apply the coupled mode theory and monitor the field amplitudes in various locations of the structure shown in Fig. S1^1^. For the coupling regions of the upper and lower waveguides, we assume identical through- and cross- coupling coefficients, $\sigma$ and $\kappa$, respectively. The end of the upper waveguide is tapered to minimize feedback; thus, its reflection coefficient is assumed to be zeroin this analysis ($c_{2}=0$). The end of the lower waveguide is sharply terminated to be used as a mirror with reflection coefficient $R$. Assuming no coupling to the neighboring ring, we set $c_{0}=d_{0}=0$. The clockwise and counterclockwise field amplitudes of the $n$-th roundtrip are denoted as $E_{n,CW}$ and $E_{n,CCW}$, and the $n+1$-th roundtrip amplitudes can be calculated from the CW and CCW fields of the $n$-th round trip. For the two coupling regions, one can write:

$\left[ \begin{matrix} a_{2} \\ c_{1} \end{matrix} \right]=\left[ \begin{matrix} \sigma& \kappa\\ \kappa& \sigma\end{matrix} \right]\left[ \begin{matrix} a_{1} \\ c_{0} \end{matrix} \right]$ (1)

$\left[ \begin{matrix} b_{4} \\ c_{3} \end{matrix} \right]=\left[ \begin{matrix} \sigma& \kappa\\ \kappa& \sigma\end{matrix} \right]\left[ \begin{matrix} b_{3} \\ c_{2} \end{matrix} \right]$ (2)

$\left[ \begin{matrix} b_{2} \\ d_{1} \end{matrix} \right]=\left[ \begin{matrix} \sigma& \kappa\\ \kappa& \sigma\end{matrix} \right]\left[ \begin{matrix} b_{1} \\ d_{0} \end{matrix} \right]$ (3)

$\left[ \begin{matrix} a_{4} \\ d_{3} \end{matrix} \right]=\left[ \begin{matrix} \sigma& \kappa\\ \kappa& \sigma\end{matrix} \right]\left[ \begin{matrix} a_{3} \\ d_{2} \end{matrix} \right]$ (4)

The parameters $E_{n,CCW}$, $a_{1}$, $a_{2}$,$a_{3}$, $E_{n,CW}$ and $b_{1}$, $b_{2}$, $b_{3}$, $d_{1}$ and $d_{2}$ are related through the following equations:


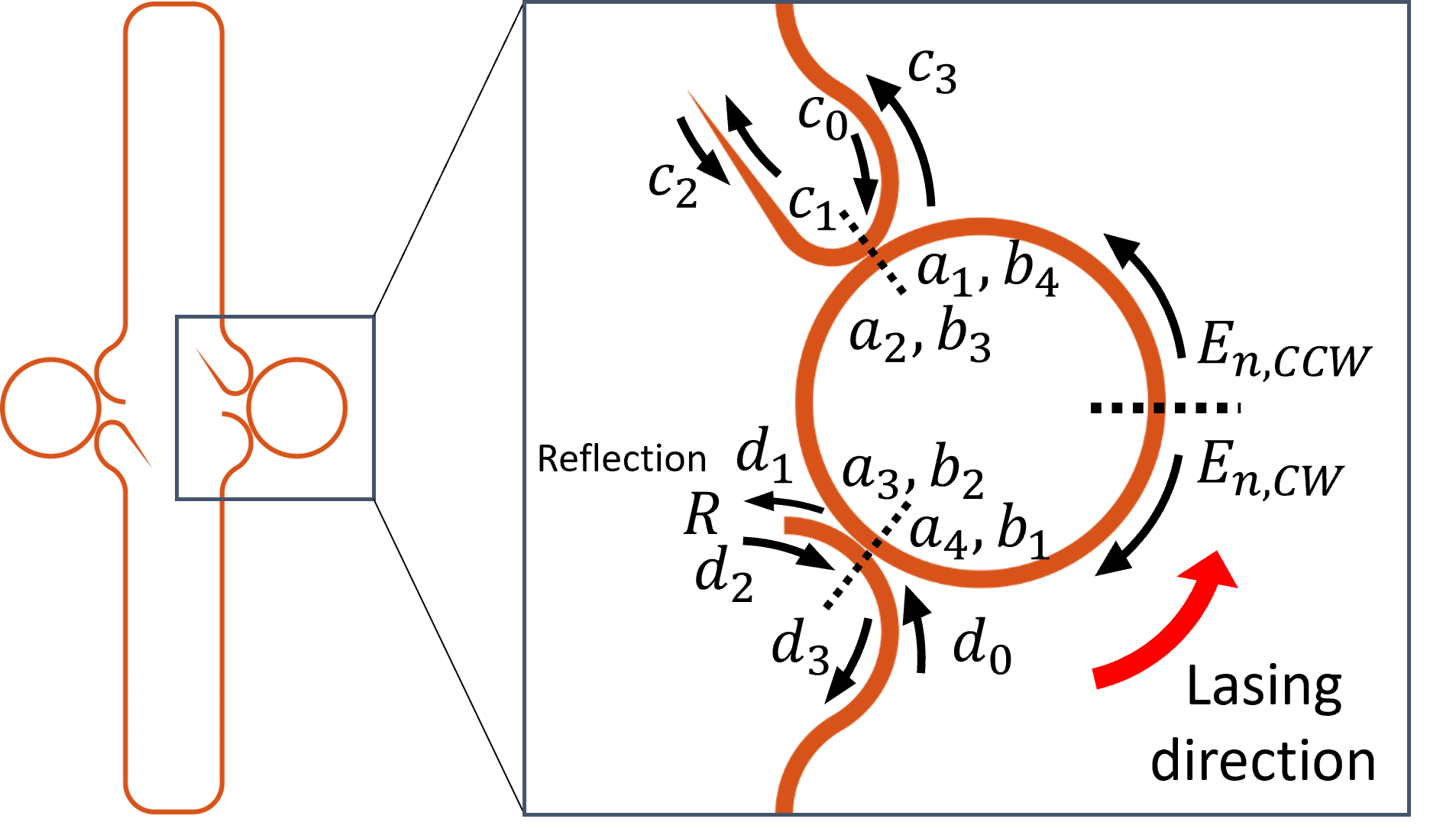


**Figure S1**. A sharply terminated waveguide at the end of the lower arm is used as a mirror to provide one-way coupling from the clockwise (CW) mode to the counterclockwise (CCW) mode in the ring resonator. The end of the waveguide on the upper arm is tapered to minimize feedback. This configuration favors counter-clockwise propagation in the ring.

$a_{1}={g_{r}}^{\frac{1}{3}}e^{-\frac{i\phi_{r}}{3}}E_{n,CCW}$ (5)

$a_{3}={g_{r}}^{\frac{1}{3}}e^{-\frac{i\phi_{r}}{3}}a_{2}$ (6)

$b_{1}={g_{r}}^{\frac{1}{3}}e^{-\frac{i\phi_{r}}{3}}E_{n,CW}$ (7)

$b_{3}={g_{r}}^{\frac{1}{3}}e^{-\frac{i\phi_{r}}{3}}b_{2}$ (8)

$d_{2}=Re^{-i2\phi_{w}}d_{1}$ (9)

where $g_{r}$ signifies the amplification/attenuation in the ring, $\phi_{r}$ is the phase accumulation in one roundtrip around the microring, and $\phi_{w}$ represents the phase accumulation due to propagation in the sharply terminated waveguide section. The CW and CCW field amplitudes of the $n+1$-th roundtrip can be written as:

$E_{n+1,CCW}={g_{r}}^{\frac{1}{3}}e^{-\frac{i\phi_{r}}{3}}a_{4}$ (10)

$E_{n+1,CW}={g_{r}}^{\frac{1}{3}}e^{-\frac{i\phi_{r}}{3}}b_{4}$ (11)

Based on the above expressions, we express the $n+1$-th roundtrip amplitudes in terms of the $n$-th roundtrip amplitudes as:

$E_{n+1,CCW}=g_{r}e^{-i\phi_{r}}\sigma^{2}E_{n+1,CCW}+R{g_{r}}^{\frac{2}{3}}e^{-\frac{i2\phi_{r}}{3}}e^{-i2\phi_{w}}\kappa^{2}E_{n+1,CW}$ (12)

$E_{n+1,CW}=g_{r}e^{-i\phi_{r}}\sigma^{2}E_{n+1,CW}$ (13)

Combining Eq. (12) and (13), we find the coupling matrix for this system as:

$\left[ \begin{aligned} E_{n+1,ccw} \\ E_{n+1,cw} \end{aligned} \right]=\left[ \begin{matrix} g_{r}e^{-i\phi_{r}}\sigma^{2} & R{g_{r}}^{\frac{2}{3}}e^{-\frac{i2\phi_{r}}{3}}e^{-i2\phi_{w}}\kappa^{2} \\ 0 & g_{r}e^{-i\phi_{r}}\sigma^{2} \end{matrix} \right]\left[ \begin{aligned} E_{n,ccw} \\ E_{n,cw} \end{aligned} \right]$ (14)

Given the evolution dynamics presented in Eq. (14), one can readily verify that the Hamiltonian associated with this coupled system is non-diagonalizable. As a result, this system only supports one mode—in this case, the mode that propagates in the counterclockwise direction. In fact, one can show that in the absence of coupling in one direction, this non-Hermitian system supports an exceptional point (EP)^2–5^. This single mode behavior is at the heart of our design for the non-Hermitian skin effect because unidirectional lasing enables the direction-dependent coupling between microring resonators.

The unidirectional behavior attributed to the presence of the one-way feedback is robust against scattering off the walls of the waveguide, and it can be designed to be broadband across several cavity modes. The analysis related to this can be found in our previous publication^3^.


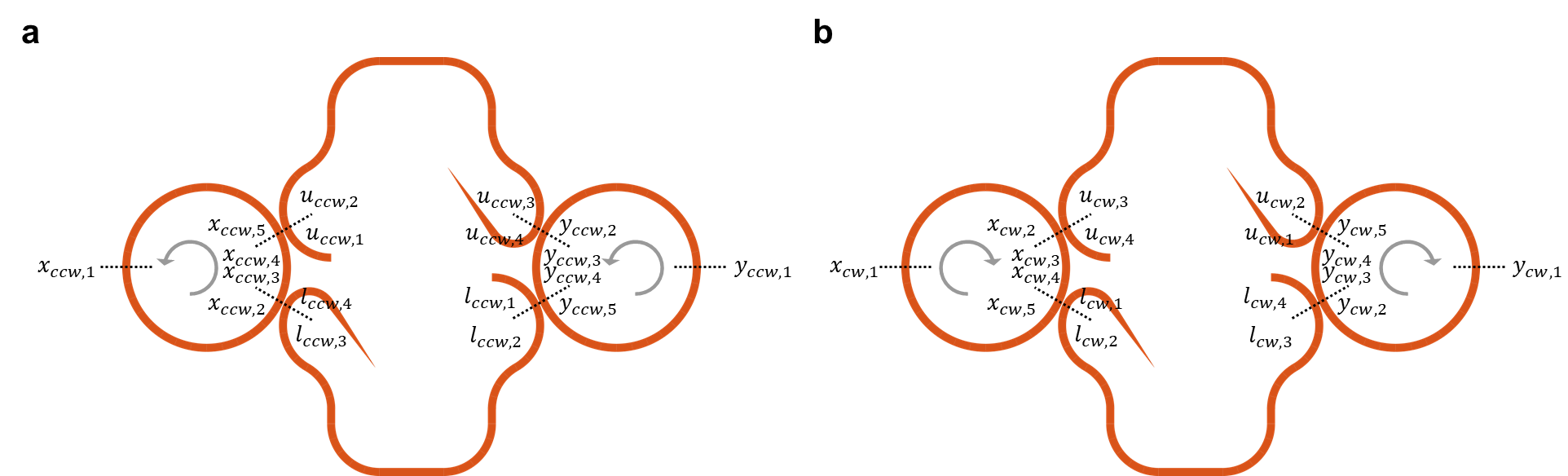


**Figure S2**. Two microring resonators coupled through two link structures with sharply terminated waveguides as reflectors. The length of the links in the figures is deliberately shrunk. Fields with opposite directions are labeled as ‘CCW’ for counterclockwise and ‘CW’ for clockwise.

To confirm that the coupled 2-element system is indeed operating in a unidirectional manner, we introduce the transfer matrices associated with the various elements involved in the links between the two resonators as shown in Fig. S2. In particular, for the four coupling regions:

$\left[ \begin{matrix} x_{ccw,3} \\ l_{ccw,4} \end{matrix} \right]=\left[ \begin{matrix} \sigma& \kappa\\ \kappa& \sigma\end{matrix} \right]\left[ \begin{matrix} x_{ccw,2} \\ l_{ccw,3} \end{matrix} \right]$ (15a)

$\left[ \begin{matrix} x_{ccw,5} \\ u_{ccw,2} \end{matrix} \right]=\left[ \begin{matrix} \sigma& \kappa\\ \kappa& \sigma\end{matrix} \right]\left[ \begin{matrix} x_{ccw,4} \\ u_{ccw,1} \end{matrix} \right]$ (15b)

$\left[ \begin{matrix} y_{ccw,3} \\ u_{ccw,4} \end{matrix} \right]=\left[ \begin{matrix} \sigma& \kappa\\ \kappa& \sigma\end{matrix} \right]\left[ \begin{matrix} y_{ccw,2} \\ u_{ccw,3} \end{matrix} \right]$ (15c)

$\left[ \begin{matrix} y_{ccw,5} \\ l_{ccw,2} \end{matrix} \right]=\left[ \begin{matrix} \sigma& \kappa\\ \kappa& \sigma\end{matrix} \right]\left[ \begin{matrix} y_{ccw,4} \\ l_{ccw,1} \end{matrix} \right]$ (15d)

$\left[ \begin{matrix} x_{cw,3} \\ u_{cw,4} \end{matrix} \right]=\left[ \begin{matrix} \sigma& \kappa\\ \kappa& \sigma\end{matrix} \right]\left[ \begin{matrix} x_{cw,2} \\ u_{cw,3} \end{matrix} \right]$ (15e)

$\left[ \begin{matrix} x_{cw,5} \\ l_{cw,2} \end{matrix} \right]=\left[ \begin{matrix} \sigma& \kappa\\ \kappa& \sigma\end{matrix} \right]\left[ \begin{matrix} x_{cw,4} \\ l_{cw,1} \end{matrix} \right]$ (15f)

$\left[ \begin{matrix} y_{cw,3} \\ l_{cw,4} \end{matrix} \right]=\left[ \begin{matrix} \sigma& \kappa\\ \kappa& \sigma\end{matrix} \right]\left[ \begin{matrix} y_{cw,2} \\ l_{cw,3} \end{matrix} \right]$ (15g)

$\left[ \begin{matrix} y_{cw,5} \\ u_{cw,2} \end{matrix} \right]=\left[ \begin{matrix} \sigma& \kappa\\ \kappa& \sigma\end{matrix} \right]\left[ \begin{matrix} y_{cw,4} \\ u_{cw,1} \end{matrix} \right]$ (15h)

For the phase accumulated during propagation in the waveguide, one can write:

$x_{ccw,2}=e^{-\frac{i\phi_{r}}{3}}x_{ccw,1}$, $x_{cw,2}=e^{-\frac{i\phi_{r}}{3}}x_{cw,1}$ (16a)

$x_{ccw,4}=e^{-\frac{i\phi_{r}}{3}}x_{ccw,3}$, $x_{cw,4}=e^{-\frac{i\phi_{r}}{3}}x_{cw,3}$ (16b)

$x_{ccw,1}=e^{-\frac{i\phi_{r}}{3}}x_{ccw,5}$, $x_{cw,1}=e^{-\frac{i\phi_{r}}{3}}x_{cw,5}$ (16c)

$y_{ccw,2}=e^{-\frac{i\phi_{r}}{3}}y_{ccw,1}$, $y_{cw,2}=e^{-\frac{i\phi_{r}}{3}}y_{cw,1}$ (16d)

$y_{ccw,4}=e^{-\frac{i\phi_{r}}{3}}y_{ccw,3}$, $y_{cw,4}=e^{-\frac{i\phi_{r}}{3}}y_{cw,3}$ (16e)

$y_{ccw,1}=e^{-\frac{i\phi_{r}}{3}}y_{ccw,5}$, $y_{cw,1}=e^{-\frac{i\phi_{r}}{3}}y_{cw,5}$ (16f)

$u_{ccw,3}=e^{-i\phi_{l}}u_{ccw,2}$, $u_{cw,3}=e^{-i\phi_{l}}u_{cw,2}$ (16g)

$l_{ccw,3}=e^{-i\phi_{l}}l_{ccw,2}$, $l_{cw,3}=e^{-i\phi_{l}}l_{cw,2}$ (16h)

$u_{ccw,1}=e^{-i2\phi_{w}}Ru_{cw,4}$, $l_{ccw,1}=e^{-i2\phi_{w}}Rl_{cw,4}$ (16i)

where $\phi_{l}$ is the phase accumulation due to propagation through the link part and $\phi_{w}$ is the phase accumulation due to propagation in the sharply terminated waveguide section. Assuming no reflection from the tapered side, we set $u_{cw,1}=l_{cw,1}=0$. Combining Eqs. 15 and Eqs. 16, one can write the transfer matrix of the systems as:

$\left[ \begin{matrix} K_{11} & K_{12} & K_{13} & K_{14} \\ K_{21} & K_{22} & K_{23} & K_{24} \\ K_{31} & K_{32} & K_{33} & K_{34} \\ K_{41} & K_{42} & K_{43} & K_{44} \end{matrix} \right]\left[ \begin{matrix} x_{ccw,1} \\ y_{ccw,1} \\ x_{cw,1} \\ y_{cw,1} \end{matrix} \right]=\hat{K}\left[ \begin{matrix} x_{ccw,1} \\ y_{ccw,1} \\ x_{cw,1} \\ y_{cw,1} \end{matrix} \right]=\Lambda\left[ \begin{matrix} x_{ccw,1} \\ y_{ccw,1} \\ x_{cw,1} \\ y_{cw,1} \end{matrix} \right]$ (17)

where $\Lambda$ is the system’s eigenvalue and $\left[ \begin{matrix} x_{ccw,1} & y_{ccw,1} & x_{cw,1} & y_{cw,1} \end{matrix} \right]^{T}$ the system’s eigenvector. The elements $K_{mn}$ in the matrix $\hat{K}$ are:

$K_{11}=-\frac{ⅇ^{ⅈ\phi_{r}}\sigma^{2}}{-1+ⅇ^{\frac{2}{3}ⅈ(3\phi_{l}+\phi_{r})}\kappa^{4}}$ (18a)

$K_{12}=-\frac{ⅇ^{ⅈ\phi_{l}+\frac{4ⅈ\phi_{r}}{3}}\kappa^{2}\sigma^{2}}{-1+ⅇ^{\frac{2}{3}ⅈ(3\phi_{l}+\phi_{r})}\kappa^{4}}$ (18b)

$K_{13}=\frac{ⅇ^{\frac{2}{3}ⅈ(\phi_{r}+3\phi_{w})}R\kappa^{2}(1+ⅇ^{\frac{4}{3}ⅈ(3\phi_{l}+\phi_{r})}\kappa^{4}{(\kappa^{2}-\sigma^{2})}^{2}+ⅇ^{\frac{2}{3}ⅈ(3\phi_{l}+\phi_{r})}(-2\kappa^{4}+2\kappa^{2}\sigma^{2}+\sigma^{4}))}{{(-1+ⅇ^{\frac{2}{3}ⅈ(3\phi_{l}+\phi_{r})}\kappa^{4})}^{2}}$ (18c)

$K_{14}=-\frac{2ⅇ^{ⅈ(\phi_{l}+\phi_{r}+2\phi_{w})}R\kappa^{2}\sigma^{2}(-1+ⅇ^{\frac{2}{3}ⅈ(3\phi_{l}+\phi_{r})}\kappa^{2}(\kappa-\sigma)(\kappa+\sigma))}{{(-1+ⅇ^{\frac{2}{3}ⅈ(3\phi_{l}+\phi_{r})}\kappa^{4})}^{2}}$ (18d)

$K_{21}=-\frac{ⅇ^{ⅈ\phi_{l}+\frac{4ⅈ\phi_{r}}{3}}\kappa^{2}\sigma^{2}}{-1+ⅇ^{\frac{2}{3}ⅈ(3\phi_{l}+\phi_{r})}\kappa^{4}}$ (19a)

$K_{22}=-\frac{ⅇ^{ⅈ\phi_{r}}\sigma^{2}}{-1+ⅇ^{\frac{2}{3}ⅈ(3\phi_{l}+\phi_{r})}\kappa^{4}}$ (19b)

$K_{23}=-\frac{2ⅇ^{ⅈ(\phi_{l}+\phi_{r}+2\phi_{w})}R\kappa^{2}\sigma^{2}(-1+ⅇ^{\frac{2}{3}ⅈ(3\phi_{l}+\phi_{r})}\kappa^{2}(\kappa-\sigma)(\kappa+\sigma))}{{(-1+ⅇ^{\frac{2}{3}ⅈ(3\phi_{l}+\phi_{r})}\kappa^{4})}^{2}}$ (19c)

$K_{24}=\frac{ⅇ^{\frac{2}{3}ⅈ(\phi_{r}+3\phi_{w})}R\kappa^{2}(1+ⅇ^{\frac{4}{3}ⅈ(3\phi_{l}+\phi_{r})}\kappa^{4}{(\kappa^{2}-\sigma^{2})}^{2}+ⅇ^{\frac{2}{3}ⅈ(3\phi_{l}+\phi_{r})}(-2\kappa^{4}+2\kappa^{2}\sigma^{2}+\sigma^{4}))}{{(-1+ⅇ^{\frac{2}{3}ⅈ(3\phi_{l}+\phi_{r})}\kappa^{4})}^{2}}$ (19d)

$K_{31}=0$ (20a)

$K_{32}=0$ (20b)

$K_{33}=-\frac{ⅇ^{ⅈ\phi_{r}}\sigma^{2}}{-1+ⅇ^{\frac{2}{3}ⅈ(3\phi_{l}+\phi_{r})}\kappa^{4}}$ (20c)

$K_{34}=-\frac{ⅇ^{ⅈ\phi_{l}+\frac{4ⅈ\phi_{r}}{3}}\kappa^{2}\sigma^{2}}{-1+ⅇ^{\frac{2}{3}ⅈ(3\phi_{l}+\phi_{r})}\kappa^{4}}$ (20d)

$K_{41}=0$ (21a)

$K_{42}=0$ (21b)

$K_{43}=-\frac{ⅇ^{ⅈ\phi_{l}+\frac{4ⅈ\phi_{r}}{3}}\kappa^{2}\sigma^{2}}{-1+ⅇ^{\frac{2}{3}ⅈ(3\phi_{l}+\phi_{r})}\kappa^{4}}$ (21c)

$K_{44}=-\frac{ⅇ^{ⅈ\phi_{r}}\sigma^{2}}{-1+ⅇ^{\frac{2}{3}ⅈ(3\phi_{l}+\phi_{r})}\kappa^{4}}$ (21d)

Because matrix elements $K_{31}=K_{32}=K_{41}=K_{42}=0$, similar to the one-way feedback case discussed above, this system provides coupling from CW modes to CCW modes but not the opposite. This system therefore only supports two CCW modes with eigenvectors:

$v_{1}=\left[ \begin{matrix} x_{ccw,1} \\ y_{ccw,1} \\ x_{cw,1} \\ y_{cw,1} \end{matrix} \right]=\left[ \begin{matrix} 1 \\ 1 \\ 0 \\ 0 \end{matrix} \right]$, and $v_{2}=\left[ \begin{matrix} x_{ccw,1} \\ y_{ccw,1} \\ x_{cw,1} \\ y_{cw,1} \end{matrix} \right]=\left[ \begin{matrix} 1 \\ -1 \\ 0 \\ 0 \end{matrix} \right]$ (22)

where $v_{1}$ represents the in-phase mode and $v_{2}$ denotes the $\pi$-out-of-phase mode. To analyze the behavior of the aforementioned coupled microring laser system and simplify the calculation, we consider a lossless, resonating, non-Hermitian system with two identical cavities and ignore the phase noise introduced by the reflector waveguide. To promote the in-phase mode, we substitute the following parameters to Eq. (17): $\phi_{r}=6q\pi$, $\phi_{w}=2p\pi$, $\phi_{l}=2m\pi$, $\kappa=0.5$, $\sigma=i\sqrt{1-{0.5}^{2}}$ and obtain the 2 eigenvalues ($q$, $p$ and $m$ are integers):

$\Lambda_{1}=1.32$, $\Lambda_{2}=0.79$ (23)

The eigenvalues associated with $v_{1}$ and $v_{2}$ are $1.32$ and $0.79$, respectively. Here, the magnitudes of the elements represent the roundtrip amplification/damping of the fields and element arguments represent the roundtrip phase change of the fields. In a laser system, the eigenmode with the highest amplification will reach the lasing threshold first under pumping. The eigenvector $v_{1}=\left[ 1 1 0 0 \right]^{T}$ represents the in-phase lasing mode under this condition.

**Section 2. Sample fabrication**


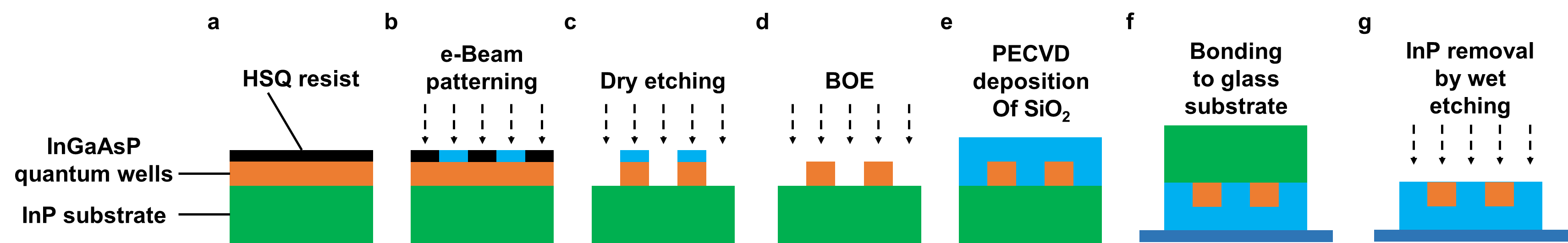


**Figure S3.** Schematic of the fabrication procedure of microring lasers. **a,** HSQ e-beam resist is spun onto the wafer. **b,** The wafer is patterned by e-beam lithography. **c,** A dry etching process to define the rings. **d,** The sample is immersed in BOE to remove the masking HSQ. **e,** A $2 \mu m$ layer of SiO_2_ is deposited via PECVD. **f,** The wafer is flipped upside-down and bonded to a glass substrate by SU-8 photoresist to provide mechanical support. **g,** Lastly, the InP substrate is wet etched by HCl.

The fabrication steps that were followed in order to realize the proposed lattices are shown in Fig. S3. Here, an XR-1541 hydrogen silsesquioxane (HSQ) solution in methyl isobutyl ketone (MIBK) is used as a negative electron beam resist. The resist is spun onto the wafer (thickness $\approx50 \mathrm{nm}$) and is soft baked at a temperature of $180^{\circ}C$ (Fig. S3a). The rings are then patterned by electron beam lithography (Fig. S3b). Next, the wafer is immersed in tetramethylammonium hydroxide (TMAH) for 120 seconds to develop the patterns, and is then rinsed in isopropyl alcohol (IPA) for 30 seconds. The HSQ that is exposed by the electron beam remains and serves as a mask for the subsequent reactive ion etching (RIE) processes. To perform the dry etching, a mixture of H_2_:CH_4_:Ar gases is used with a ratio of $20:10:4 \mathrm{sccm}$. The sample is biased at -450V and the chamber pressure was held at $75 \mathrm{mT}$ (Fig. S3c). The wafer is then cleaned with oxygen plasma to remove organic contaminations and polymers that form during the dry etching process (O_2_: $20\mathrm{sccm}$ flow, sample bias: -300V, chamber pressure: $125 \mathrm{mT}$). The patterns are then submerged in buffered oxide etch (BOE) for 15 seconds to remove the HSQ mask (Fig. S3d). After this, a $2 \mu m$ layer of SiO_2_ is deposited onto the wafer using plasma-enhanced chemical vapor deposition (PECVD) (Fig. S3e). We used SU-8 2010 photoresist to bond the wafer to a glass substrate for mechanical support (Fig. S3f). After spinning the photoresist, the sample is placed on the glass, pattern-side-down, and exposed for 30 seconds on both sides. Lastly, the remaining InP substrate is entirely removed by wet etching in hydrochloric acid (HCl) for 40 minutes (Fig. S3g)^6^.

**Section 3. Mode discrimination via complex asymmetric coupling**

For the 2-element system described in the manuscript (Fig. 2a), one can write its Hamiltonian as:

$\hat{H}=\left[ \begin{matrix} \omega_{0} & i\gamma_{l}e^{i\beta L} \\ i\gamma_{u}e^{i\beta L} & \omega_{0} \end{matrix} \right]$ (22)

where $\omega_{0}$ is the resonance frequency of each resonator in the absence of coupling, $\beta$ is the propagation constant of the TE_0_ mode of the waveguide, $L$ is the length of the links, and $\gamma_{u}$, $\gamma_{l}$ are the gain/loss coefficients that are in turn controlled by the pumping profiles of the upper and lower links, respectively. One can easily find the eigenvalues $\omega_{1,2}=\omega_{0}\pm i\sqrt{\gamma_{u}\gamma_{l}}e^{i\beta L}$ and their corresponding eigenvectors $[1 \pm\sqrt{\gamma_{u}/\gamma_{l}}]$. Notice that when $\beta L=m\pi+\pi/2$, the two eigenvalues are $\omega_{1,2}=\omega_{0}\mp\sqrt{\gamma_{u}\gamma_{l}}$ ($\omega_{1,2}=\omega_{0}\pm\sqrt{\gamma_{u}\gamma_{l}}$) for an even (odd) integer number $m$. This indicates that the system undergoes a frequency splitting behavior without a mode selection effect. However, when $\beta L=m\pi$, the two eigenvalues become $\omega_{1,2}=\omega_{0}\pm i\sqrt{\gamma_{u}\gamma_{l}}$ ($\omega_{1,2}=\omega_{0}\mp i\sqrt{\gamma_{u}\gamma_{l}}$) for an even (odd) integer number $m$. This introduces an imaginary part to the eigenvalues, which is acting as gain/loss that alter the quality factor of the modes. The mode that has a positive imaginary part will have a higher quality factor and reach the lasing threshold first while the mode that has a negative imaginary part will be suppressed. By properly designing the length of the links $L$, one can achieve mode selection in such a system with complex coupling.

In experiments, to ensure the eigenvalues are imaginary and the systems are operating in a single mode, we design the length of the links to be $L=266\pi/\beta=94.76 \mu m$. Knowing that fabrication uncertainties and environmental perturbations may alter the experiment results, we fabricated 16 samples with sweeping link lengths from $L_{1}=266\pi/\beta=94.76 \mu m$ to $L_{16}=267\pi/\beta=95.12 \mu m$. By testing samples with different link length, we find the sample with link length that satisfies the phase condition. Examining all 16 samples can indicate if the $\beta L$ is near $m\pi$ or near $m\pi+\pi/2$. The experiment results in the manuscript indeed show that the system is single moded when $\beta L$ is near $m\pi$ (Figs. 2,3), and multi-moded when $\beta L$ is near $m\pi+\pi/2$ (Fig. 4).

**Section 4. Characterization setup**


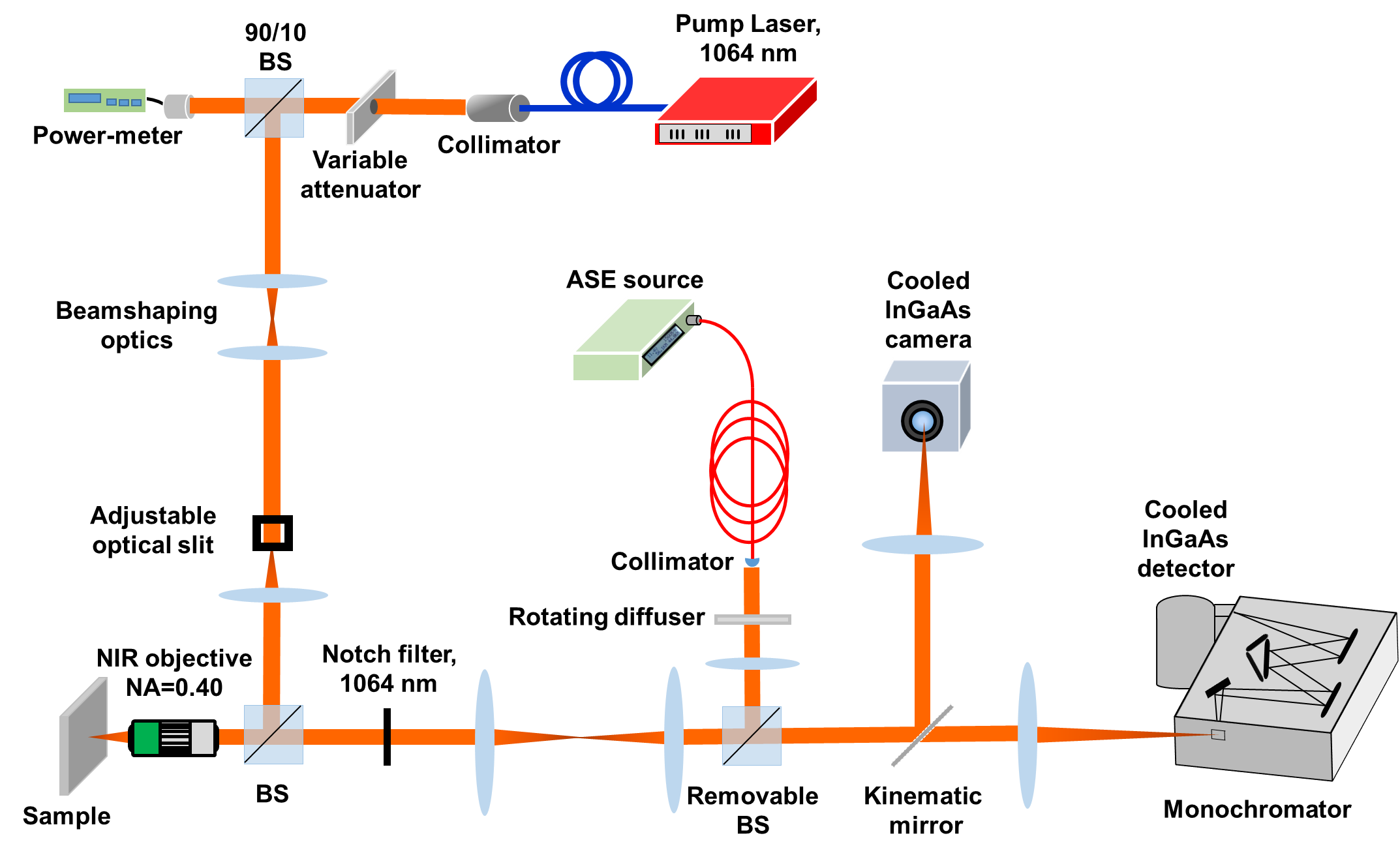


**Figure S4**. Schematic of the μ-PL characterization setup. The microrings are pumped by a pulsed laser ($15 \mathrm{ns}$ pulse width, $290 \mathrm{kHz}$ repetition rate). The pump beam is focused onto the sample with a 20$\times$ objective, and this objective in turn also collects the emission from the samples. Light is then either directed to a linear array detector for spectral measurements or to an IR camera for intensity profile observation.

A micro-photoluminescence (μ-PL) setup, depicted in Fig. S4, is used to characterize the structures. The sample is optically pumped by a pulsed (duration: $15 ns$, repetition rate: $290 kHz$) laser operating at a wavelength of $1064 nm$ (SPI fiber laser). A beam shaping system with additional metal mask and knife edges is designed to realize the desired pump size/shape. A $20\times$ microscope objective ($NA: 0.40$) is used to project the pump beam on the structure and to collect the photoluminescent emission from the sample. The surface of the sample is imaged by two cascaded 4-f imaging systems into an IR camera (Indigo). A broadband amplified spontaneous emission (ASE) device is used to illuminate the sample in order to properly position the pump beam with respect to the pattern. A notch filter is placed in the path of emission to attenuate the pump beam. The output spectra are obtained by a monochromator (Horiba) equipped with an attached linear array InGaAs detector (Synapse CCD).

**Section 5. Data of continuously changed pump profiles on 5-element systems**

Additional data for continuously changing pump profiles are shown in Fig. S5 (see also the Supplementary Videos 1 and 2). From Fig. S5a to Fig. S5e, and from Fig. S5f to Fig. S5j, the pumping profiles are shifted continuously from upper links to lower links. In this case, the asymmetric coupling ratio $\gamma_{u}/\gamma_{l}$ is continuously lowered from its maximum to its minimum, directing the non-Hermitian skin effect from right to left.


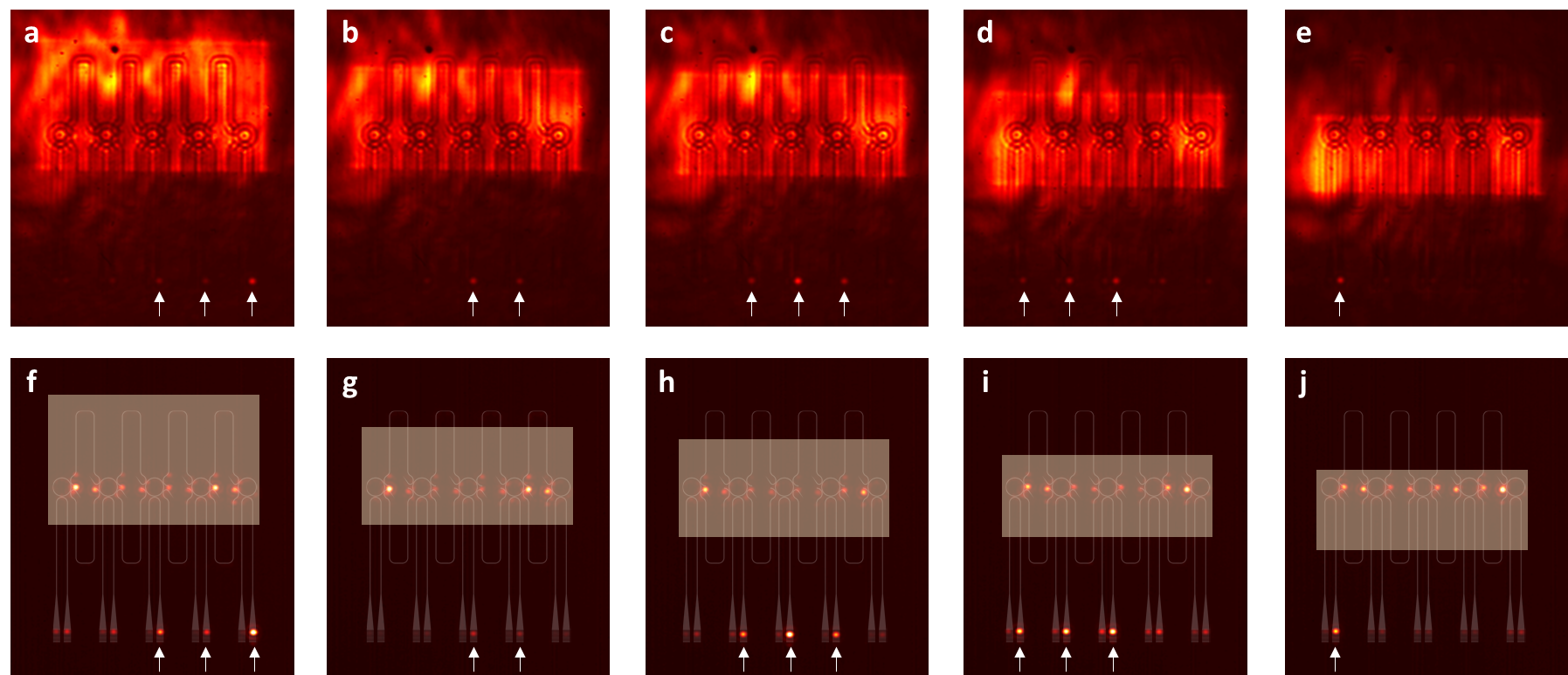


**Figure S5**. Intensity profiles of the 5-element systems showing shifting of direction of the non-Hermitian skin effect by continuously changing the pumping scheme. **a-e,** Intensity profiles taken without the 1064 nm notch filter. The bright rectangular region is pumped. **f-j,** Intensity profiles taken with the 1064 nm notch filter. Pump regions are marked by white rectangles and structures are marked by schematics.

**Section 6. Data of 11-element lattices**

Figure S6 shows the additional data of 11-element lattices. The results are similar to the results of 2-element and 5-element systems, where the non-Hermitian skin effect can be demonstrated and controlled by selectively pumping the upper or lower links.


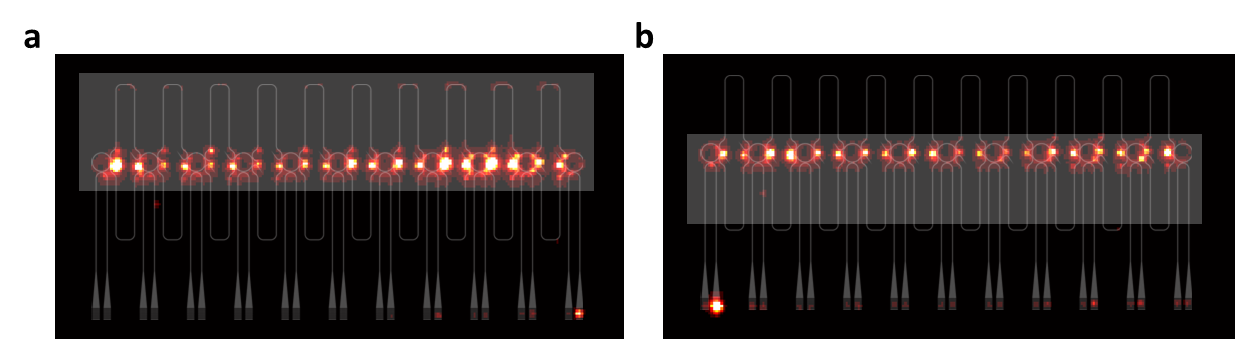


**Figure S6**. Intensity profiles of non-Hermitian skin effect in 11-element systems. Pump regions are marked by white rectangles and structures are marked by schematics. **a,** When the upper links are pumped and the lower links are left unpumped, the maximum output appears at the right end of the lattice. **b,** When the upper links are unpumped while the lower links are pumped, the skin effect is redirected to the left end of the system.

**Section 7. Spatial coupled mode theory analysis for wavelength-dependency in coupling phase**

In order to properly account for the phase variation in the links, one needs to incorporate that in the model and calculate the resonant frequency and magnitude of the modes accordingly. Here we provide the detailed analysis for the case of two resonators, as shown in Fig. S7.

In order to account for link phase variation with wavelength, we formulate the problem based on spatial coupled mode theory that relates the fields $a_{0}$ and $b_{0}$ to the fields at the same locations after one round trip in the cavity labeled as $a_{4}$ and $b_{4}$, respectively.


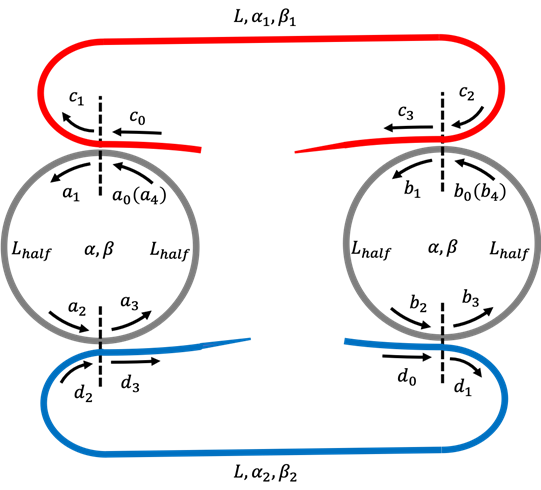


**Figure S7**. 2-element microring laser system that supports multimode operations. Microring lasers are assumed to support CCW mode only.

For the 4 coupling regions between the rings and the links, the coupling matrix is:

$M_{1}=\left[ \begin{matrix} \sigma& \kappa\\ \kappa& \sigma\end{matrix} \right]$ (23)

where $\sigma$ and $\kappa$ are the through- and cross- coupling coefficients, respectively. The field amplitudes are related through the following equations:

$\left[ \begin{matrix} a_{1} \\ c_{1} \end{matrix} \right]=M_{1}\cdot\left[ \begin{matrix} a_{0} \\ c_{0} \end{matrix} \right]$, $a_{2}=e^{\left( \alpha+j\beta\right)L_{half}}a_{1}$, (24a)

$\left[ \begin{matrix} a_{3} \\ d_{3} \end{matrix} \right]=M_{1}\cdot\left[ \begin{matrix} a_{2} \\ d_{2} \end{matrix} \right]$, $a_{4}=e^{\left( \alpha+j\beta\right)L_{half}}a_{3}$, (24b)

$\left[ \begin{matrix} b_{1} \\ c_{3} \end{matrix} \right]=M_{1}\cdot\left[ \begin{matrix} b_{0} \\ c_{2} \end{matrix} \right]$, $b_{2}=e^{\left( \alpha+j\beta\right)L_{half}}b_{1}$, (24c)

$\left[ \begin{matrix} b_{3} \\ d_{1} \end{matrix} \right]=M_{1}\cdot\left[ \begin{matrix} b_{2} \\ d_{0} \end{matrix} \right]$, $b_{4}=e^{\left( \alpha+j\beta\right)L_{half}}b_{3}$, (24d)

$c_{2}=e^{\left( \alpha_{1}+j\beta_{1} \right)L}c_{1}$, $d_{2}=e^{\left( \alpha_{2}+j\beta_{2} \right)L}d_{1}$, (24e)

The resulting transfer matrix is expressed as follows:

$\left[ \begin{matrix} a_{4} \\ b_{4} \end{matrix} \right]=e^{2\left( j\beta+\alpha\right)L_{half}}\left[ \begin{matrix} \sigma^{2}+\kappa^{4}e^{\left( j\beta_{1}+\alpha_{1} \right)L}e^{\left( j\beta_{2}+\alpha_{2} \right)L} & \kappa^{2}\sigma e^{\left( j\beta_{2}+\alpha_{2} \right)L} \\ \kappa^{2}\sigma e^{\left( j\beta_{1}+\alpha_{1} \right)L} & \sigma^{2} \end{matrix} \right]\left[ \begin{matrix} a_{0} \\ b_{0} \end{matrix} \right]$ (25)

The eigenvalues of the system are given by:

$\lambda_{1,2}=\frac{1}{2}e^{2\left( j\beta+\alpha\right)L_{half}}\left( \begin{aligned} \kappa^{4}e^{\left( \alpha_{1}+\alpha_{2}+j\beta_{1}+j\beta_{2} \right)L}+2\sigma^{2} \\ \pm\sqrt{\kappa^{8}e^{2\left( \alpha_{1}+\alpha_{2}+j\beta_{1}+j\beta_{2} \right)L}+4\kappa^{4}\sigma^{2}e^{\left( \alpha_{1}+\alpha_{2}+j\beta_{1}+j\beta_{2} \right)L}} \end{aligned} \right)$ (26)

Similarly, the corresponding eigenvectors are as follows:

$V_{1,2}=\left[ \frac{e^{-\left( j\beta_{1}+\alpha_{1} \right)L}}{2\kappa^{2}\sigma}\left( \begin{aligned} \kappa^{4}e^{\left( \alpha_{1}+\alpha_{2}+j\beta_{1}+j\beta_{2} \right)L} \\ \pm\sqrt{\kappa^{8}e^{2\left( \alpha_{1}+\alpha_{2}+j\beta_{1}+j\beta_{2} \right)L}+{4\kappa}^{4}\sigma^{2}e^{\left( \alpha_{1}+\alpha_{2}+j\beta_{1}+j\beta_{2} \right)L}} \end{aligned} \right) 1 \right]^{T}$ (27)

In order to find the resonance frequency of the modes, one must find the wavelength at which the phase of the eigenvalues becomes zero. The magnitude of the eigenvalue gives the gain/loss of the mode.


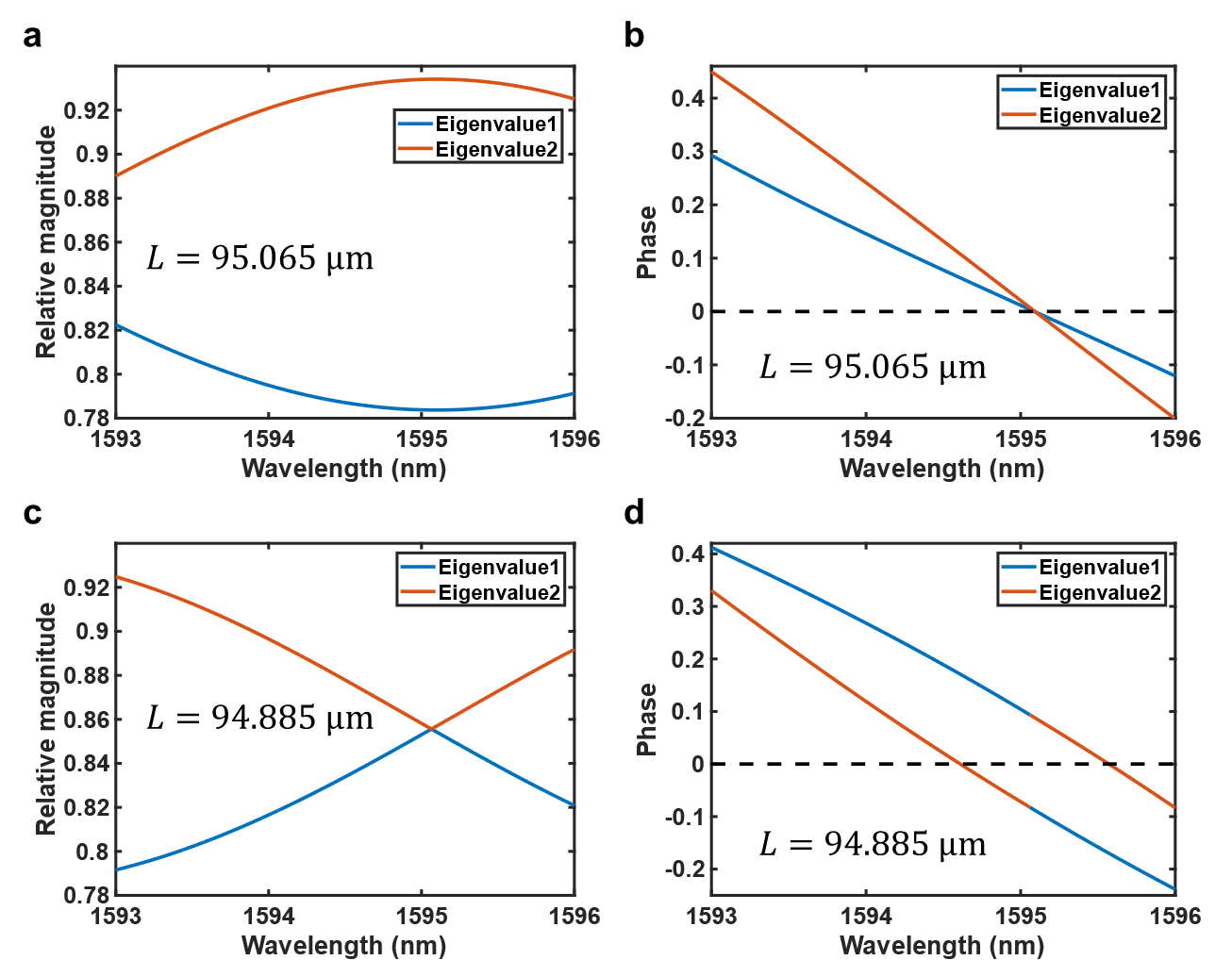


**Figure S8**. Relative magnitude and phase of the eigenvalues for two different link lengths apart by $\pi/2$ phase shift. For (a) and (b), $L=95.065 \mu m$. For (c) and (d), $L=94.885 \mu m$.

Figure. S8 shows the associated phase and magnitude of the eigenvalues for two values of $L$ (specifically when $L=95.065 \mu m$ and the other case is $L=94.885 \mu m$) and as a function of wavelength. Given that $n_{eff}=2.24$, at a wavelength of $\lambda=1.595 \mu m$, this length difference results in $\sim\pi/2$ phase shift. When $L=95.065 \mu m$ (Fig. S8a,b), corresponding to a $m\pi$ phase condition, the two modes are lasing at the same wavelength, with varying magnitude of the eigenvalues. Since the modes are at the same frequency, the one with the higher magnitude of eigenvalue is going to lase, thus leading to single mode lasing. On the other hand, at $L=94.885 \mu m$(Fig. S8c,d) which corresponds to a phase condition of $m\pi+\pi/2$, two modes appear that satisfy the resonant condition while being $\sim1 \mathrm{nm}$ apart, both having a very similar magnitude- therefore they are both lasing.

**Section 8. Gain-dependency in coupling phase**

As the link structures are pumped with different profiles during the experiments, corresponding carrier density change may introduce phase difference in the coupling terms. The change in the phase due to excess carrier density follows:

$\Delta\phi=\frac{\alpha}{2}\Delta gL$ (28)

where $\Delta\phi$ is the gain induced phase change. $\alpha$ is the linewidth enhancement factor and $\Delta g$ is the change in gain. For our experiments, the value of gain is $\cong50 cm^{-1}$, the loss is $\cong-450 cm^{-1}$ and the linewidth enhancement factor is $2$. The resulting phase from the fully pumped link is $\Delta\phi=0.5$ and for the partially pumped link it is $\Delta\phi=0.15$. This results in a net change of $\Delta\phi=0.35$ or roughly $\pi/10$ phase difference. This phase difference minimally affects the performance of the system. The eigenvalues as calculated above with this extra phase are given in Fig. S9.


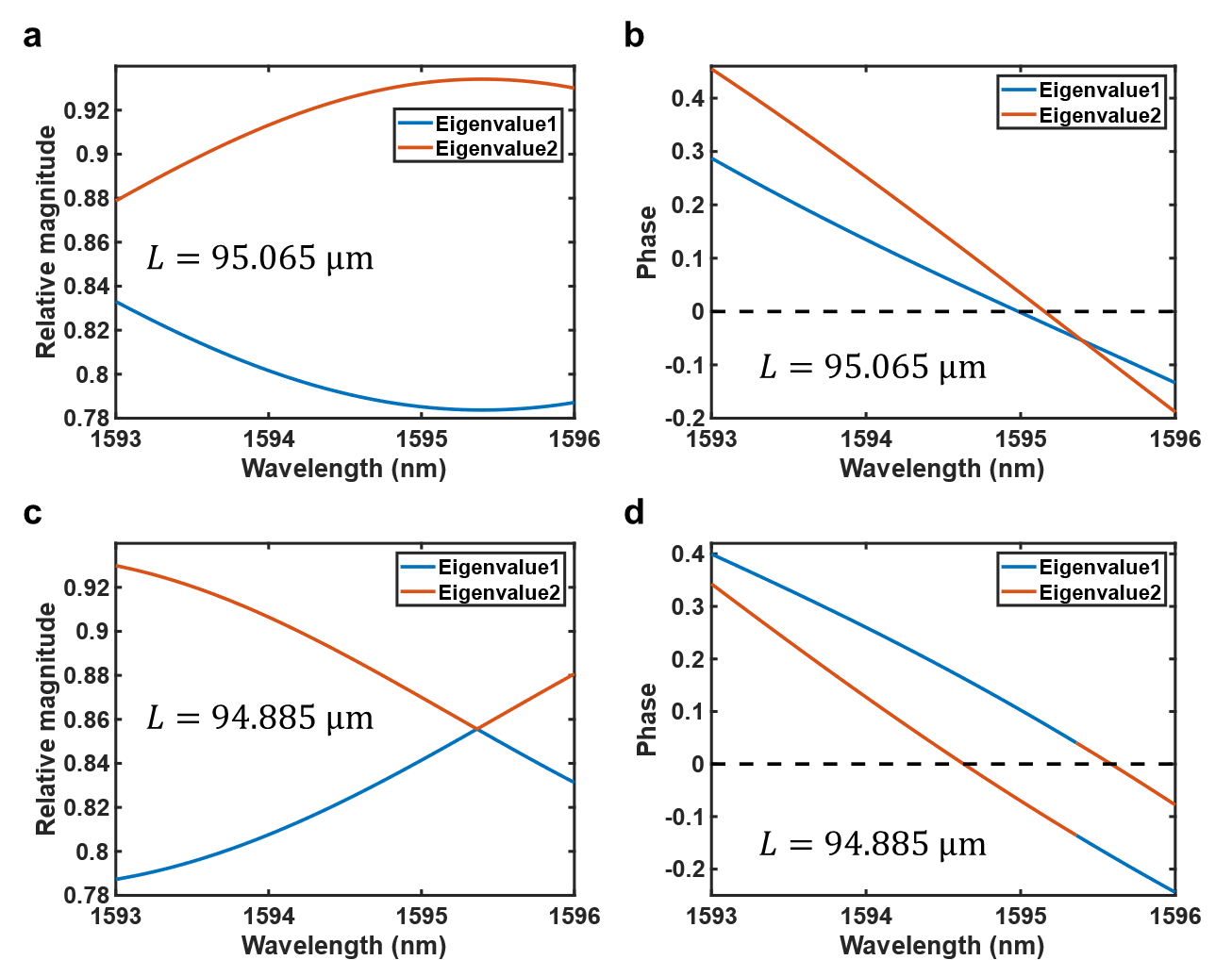


**Figure S9**. Relative magnitude and phase of the eigenvalues for two different link lengths apart by $\pi/2$ phase shift with gain induced phase change. For (a) and (b), $L=95.065 \mu m$. For (c) and (d), $L=94.885 \mu m$

As it can be seen in Fig. S9, for the case $m\pi$ phase condition (Fig. S9a,b), the system now supports two modes with very small frequency separation, but one of the eigenvalues has a considerably smaller magnitude. For the case of $m\pi+\pi/2$ (Fig. S9c,d) no discernable difference is observed.

**Section 9. Spatial coupled mode theory analysis for 5-element systems**

To analyze the dispersion of the coupling phase when the 5-element microring laser array is operating in multimode condition, we apply the spatial coupled mode theory and monitor the field amplitudes in various locations of the structure shown in Fig. S10.


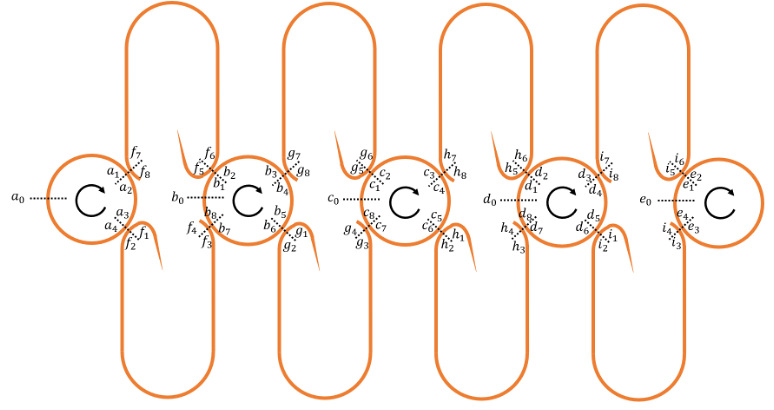


**Figure S10**. 5-element microring laser array that supports multimode operations. Microring lasers are assumed to support CW mode only.

For the 16 coupling regions between the rings and the links, the coupling matrix is:

$M_{1}=\left[ \begin{matrix} \sigma& \kappa\\ \kappa& \sigma\end{matrix} \right]$ (29)

where $\sigma$ and $\kappa$ are the through- and cross- coupling coefficients, respectively. The field amplitudes are related through the following equations:

$a_{1}=e^{-\frac{i\phi_{r}}{3}}a_{0}$, $\left[ \begin{matrix} a_{2} \\ f_{8} \end{matrix} \right]=M_{1}\cdot\left[ \begin{matrix} a_{1} \\ f_{7} \end{matrix} \right]$, $a_{3}=e^{-\frac{i\phi_{r}}{3}}a_{2}$, $\left[ \begin{matrix} a_{4} \\ f_{2} \end{matrix} \right]=M_{1}\cdot\left[ \begin{matrix} a_{3} \\ f_{1} \end{matrix} \right]$, $a_{0^{'}}=e^{-\frac{i\phi_{r}}{3}}a_{4}$; (30a)

$b_{1}=e^{-\frac{i\phi_{r}}{6}}b_{0}$, $\left[ \begin{matrix} b_{2} \\ f_{6} \end{matrix} \right]=M_{1}\cdot\left[ \begin{matrix} b_{1} \\ f_{5} \end{matrix} \right]$, $b_{3}=e^{-\frac{i\phi_{r}}{6}}b_{2}$, $\left[ \begin{matrix} b_{4} \\ g_{8} \end{matrix} \right]=M_{1}\cdot\left[ \begin{matrix} b_{3} \\ g_{7} \end{matrix} \right]$, $b_{5}=e^{-\frac{i\phi_{r}}{3}}b_{4}$, (30b)

$\left[ \begin{matrix} b_{6} \\ g_{2} \end{matrix} \right]=M_{1}\cdot\left[ \begin{matrix} b_{5} \\ g_{1} \end{matrix} \right]$, $b_{7}=e^{-\frac{i\phi_{r}}{6}}b_{6}$, $\left[ \begin{matrix} b_{8} \\ f_{4} \end{matrix} \right]=M_{1}\cdot\left[ \begin{matrix} b_{7} \\ f_{3} \end{matrix} \right]$, $b_{0^{'}}=e^{-\frac{i\phi_{r}}{6}}b_{8}$; (30c)

$c_{1}=e^{-\frac{i\phi_{r}}{6}}c_{0}$, $\left[ \begin{matrix} c_{2} \\ g_{6} \end{matrix} \right]=M_{1}\cdot\left[ \begin{matrix} c_{1} \\ g_{5} \end{matrix} \right]$, $c_{3}=e^{-\frac{i\phi_{r}}{6}}c_{2}$, $\left[ \begin{matrix} c_{4} \\ h_{8} \end{matrix} \right]=M_{1}\cdot\left[ \begin{matrix} c_{3} \\ h_{7} \end{matrix} \right]$, $c_{5}=e^{-\frac{i\phi_{r}}{3}}c_{4}$, (30d)

$\left[ \begin{matrix} c_{6} \\ h_{2} \end{matrix} \right]=M_{1}\cdot\left[ \begin{matrix} c_{5} \\ h_{1} \end{matrix} \right]$, $c_{7}=e^{-\frac{i\phi_{r}}{6}}c_{6}$, $\left[ \begin{matrix} c_{8} \\ g_{4} \end{matrix} \right]=M_{1}\cdot\left[ \begin{matrix} c_{7} \\ g_{3} \end{matrix} \right]$, $c_{0^{'}}=e^{-\frac{i\phi_{r}}{6}}c_{8}$; (30e)

$d_{1}=e^{-\frac{i\phi_{r}}{6}}d_{0}$, $\left[ \begin{matrix} d_{2} \\ h_{6} \end{matrix} \right]=M_{1}\cdot\left[ \begin{matrix} d_{1} \\ h_{5} \end{matrix} \right]$, $d_{3}=e^{-\frac{i\phi_{r}}{6}}d_{2}$, $\left[ \begin{matrix} d_{4} \\ i_{8} \end{matrix} \right]=M_{1}\cdot\left[ \begin{matrix} d_{3} \\ i_{7} \end{matrix} \right]$, $d_{5}=e^{-\frac{i\phi_{r}}{3}}d_{4}$, (30f)

$\left[ \begin{matrix} d_{6} \\ i_{2} \end{matrix} \right]=M_{1}\cdot\left[ \begin{matrix} d_{5} \\ i_{1} \end{matrix} \right]$, $d_{7}=e^{-\frac{i\phi_{r}}{6}}d_{6}$, $\left[ \begin{matrix} d_{8} \\ h_{4} \end{matrix} \right]=M_{1}\cdot\left[ \begin{matrix} d_{7} \\ h_{3} \end{matrix} \right]$, $d_{0^{'}}=e^{-\frac{i\phi_{r}}{6}}d_{8}$; (30g)

$e_{1}=e^{-\frac{i\phi_{r}}{6}}e_{0}$, $\left[ \begin{matrix} e_{2} \\ i_{6} \end{matrix} \right]=M_{1}\cdot\left[ \begin{matrix} e_{1} \\ i_{5} \end{matrix} \right]$, $e_{3}=e^{-\frac{i2\phi_{r}}{3}}e_{2}$, $\left[ \begin{matrix} e_{4} \\ i_{4} \end{matrix} \right]=M_{1}\cdot\left[ \begin{matrix} e_{3} \\ i_{3} \end{matrix} \right]$, $e_{0^{'}}=e^{-\frac{i\phi_{r}}{6}}e_{4}$; (30h)

$f_{3}=e^{-i\phi_{l}+\alpha_{l}L}f_{2}$, $f_{7}=e^{-i\phi_{u}+\alpha_{u}L}f_{6}$, $g_{3}=e^{-i\phi_{l}+\alpha_{l}L}g_{2}$, $g_{7}=e^{-i\phi_{u}+\alpha_{u}L}g_{6}$, (30i)

$h_{3}=e^{-i\phi_{l}+\alpha_{l}L}h_{2}$, $h_{7}=e^{-i\phi_{u}+\alpha_{u}L}h_{6}$, $i_{3}=e^{-i\phi_{l}+\alpha_{l}L}i_{2}$, $i_{7}=e^{-i\phi_{u}+\alpha_{u}L}i_{6}$ . (30j)

$\phi_{r}=2\pi r\beta+ 2\pi ri\alpha_{r}$ is the phase accumulation and amplification in one roundtrip around the microring. $\phi_{u}=\beta L$ and $\phi_{l}=\beta L$ are the phase accumulation of the upper and lower links. $\alpha_{u}$ and $\alpha_{l}$ are the gain/loss factor of the upper and lower links, respectively. The transfer matrix of the system can be written as:

$\left[ \begin{matrix} A_{11} & A_{12} & 0 & 0 & 0 \\ A_{21} & A_{22} & A_{23} & 0 & 0 \\ 0 & A_{32} & A_{33} & A_{34} & 0 \\ 0 & 0 & A_{43} & A_{44} & A_{45} \\ 0 & 0 & 0 & A_{54} & A_{55} \end{matrix} \right]\left[ \begin{matrix} a_{0} \\ b_{0} \\ c_{0} \\ d_{0} \\ e_{0} \end{matrix} \right]=\hat{A}\left[ \begin{matrix} a_{0} \\ b_{0} \\ c_{0} \\ d_{0} \\ e_{0} \end{matrix} \right]=\Lambda\left[ \begin{matrix} a_{0} \\ b_{0} \\ c_{0} \\ d_{0} \\ e_{0} \end{matrix} \right]$ (31)

where $\Lambda$ is the system’s eigenvalue and $\left[ \begin{matrix} a_{0} & b_{0} & c_{0} & d_{0} & e_{0} \end{matrix} \right]^{T}$ the system’s eigenvector. The elements $A_{mn}$ in the matrix $\hat{A}$ are:

$A_{11}=ⅇ^{2\pi r(\alpha_{r}+ⅈ\beta)}\sigma^{2}$ (32a)

$A_{12}=ⅇ^{\frac{5}{3}\pi r\left( \alpha_{r}+i\beta\right)+L(\alpha_{u}+i\beta)}\kappa^{2}\sigma$ (32b)

$A_{21}=ⅇ^{\frac{5}{3}\pi r\left( \alpha_{r}+i\beta\right)+L(\alpha_{l}+i\beta)}\kappa^{2}\sigma$ (32c)

$A_{22}=ⅇ^{\frac{4}{3}\pi r(\alpha_{r}+i\beta)+(\alpha_{u}+\alpha_{l}+2ⅈ\beta)L}\kappa^{4}+e^{2\pi r(\alpha_{r}+i\beta)}\sigma^{4}$ (32d)

$A_{23}=ⅇ^{\frac{5}{3}\pi r\left( \alpha_{r}+i\beta\right)+L\left( \alpha_{u}+i\beta\right)}\kappa^{2}\sigma^{2}$ (32e)

$A_{32}=ⅇ^{\frac{5}{3}\pi r\left( \alpha_{r}+i\beta\right)+L(\alpha_{l}+i\beta)}\kappa^{2}\sigma^{2}$ (32f)

$A_{33}=ⅇ^{\frac{4}{3}\pi r(\alpha_{r}+i\beta)+(\alpha_{u}+\alpha_{l}+2ⅈ\beta)L}\kappa^{4}+e^{2\pi r(\alpha_{r}+i\beta)}\sigma^{4}$ (32g)

$A_{34}=ⅇ^{\frac{5}{3}\pi r\left( \alpha_{r}+i\beta\right)+L\left( \alpha_{u}+i\beta\right)}\kappa^{2}\sigma^{2}$ (32h)

$A_{43}=ⅇ^{\frac{5}{3}\pi r\left( \alpha_{r}+i\beta\right)+L(\alpha_{l}+i\beta)}\kappa^{2}\sigma^{2}$ (32i)

$A_{44}=ⅇ^{\frac{4}{3}\pi r(\alpha_{r}+i\beta)+(\alpha_{u}+\alpha_{l}+2ⅈ\beta)L}\kappa^{4}+e^{2\pi r(\alpha_{r}+i\beta)}\sigma^{4}$ (32j)

$A_{45}=ⅇ^{\frac{5}{3}\pi r\left( \alpha_{r}+i\beta\right)+L\left( \alpha_{u}+i\beta\right)}\kappa^{2}\sigma^{2}$ (32k)

$A_{54}=ⅇ^{\frac{5}{3}\pi r\left( \alpha_{r}+i\beta\right)+L(\alpha_{l}+i\beta)}\kappa^{2}\sigma^{2}$ (32l)

$A_{55}=ⅇ^{\frac{4}{3}\pi r(\alpha_{r}+i\beta)+(\alpha_{u}+\alpha_{l}+2ⅈ\beta)L}\kappa^{4}+e^{2\pi r(\alpha_{r}+i\beta)}\sigma^{4}$ (32m)

Because of the complexity of the analytical form for the eigenvalues of the transfer matrix, here we only focus on the numerical solution of the eigenvalues. Figure S11 shows the phase and magnitude as a function of wavelength for two values of $L$ (specifically when $L=95.065 \mu m$ (resulting in $\beta L\cong m\pi$) and the other case is $L=94.885 \mu m$ (where $\beta L\cong(m+0.5)\pi$ )).

When $L=95.065 \mu m$ (Fig. S11a,b), corresponding to a $m\pi$ phase condition, the five modes are lasing at the same wavelength, with varying magnitude of the eigenvalues. Since the modes are at the same frequency, only the one with the largest eigenvalue will lase, thus leading to a single mode lasing operation.

On the other hand, when $L=94.885 \mu m$ (Fig. S11c,d) which corresponds to a phase condition of $m\pi+\pi/2$, five modes appear that satisfy the resonant condition while being $\sim2 nm$ apart, all having a very similar magnitude (marked by red circles in Fig. 11c), thus leading to multimode lasing. Since the splitting between individual modes is less than the resolution of our spectrometer (0.64 nm), the multi-mode spectra shown in the manuscript does not resolve all the modes.


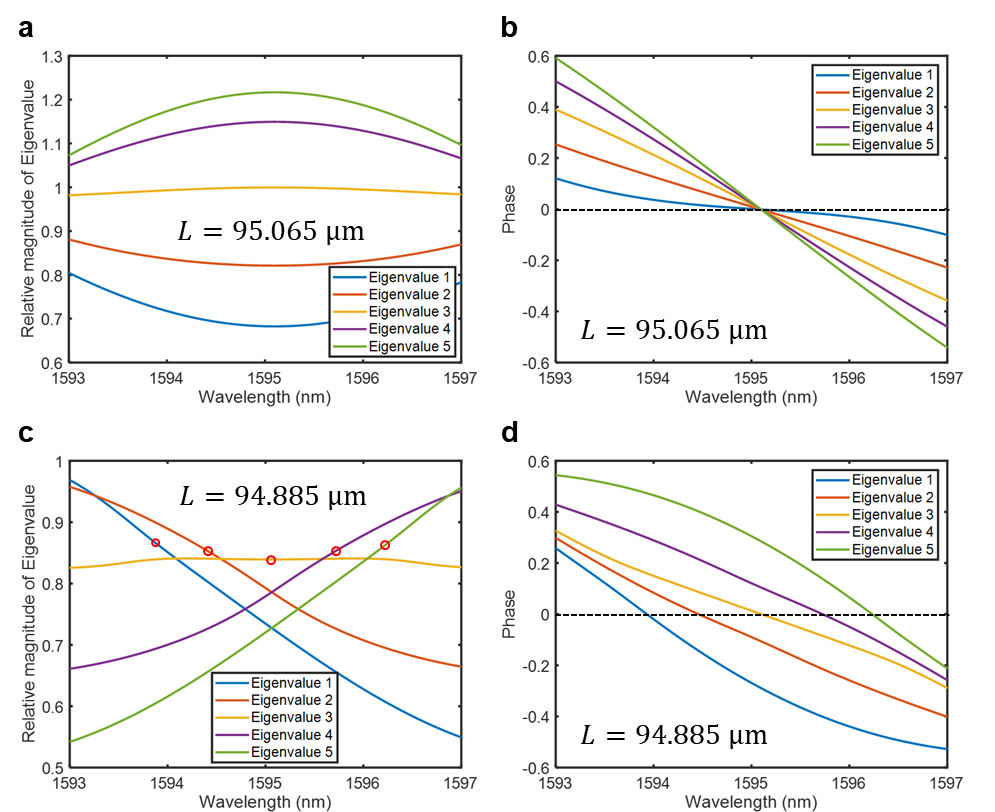


**Figure S11.** Relative magnitude and phase of the eigenvalues for two different link lengths apart by $\pi/2$ phase shift. For (a) and (b), $L=95.065 \mu m$. For (c) and (d), $L=94.885 \mu m$. Red circles in (c) indicate the resonances of individual modes and 5 modes have similar magnitudes.

References:

1. Haus, H. A. *Waves and Fields in Optoelectronics*. (Prentice-Hall, 1984).

2. Ren, J. *et al.* Unidirectional light emission in PT-symmetric microring lasers. *Opt. Express* **26**, 27153–27160 (2018).

3. Hayenga, W. E. *et al.* Direct Generation of Tunable Orbital Angular Momentum Beams in Microring Lasers with Broadband Exceptional Points. *ACS Photonics* **6**, 1895–1901 (2019).

4. Zhong, Q. *et al.* Sensing with Exceptional Surfaces in Order to Combine Sensitivity with Robustness. *Phys. Rev. Lett.* **122**, 153902 (2019).

5. Soleymani, S. *et al.* Chiral and degenerate perfect absorption on exceptional surfaces. *Nat Commun* **13**, 599 (2022).

6. Hodaei, H., Miri, M.-A., Heinrich, M., Christodoulides, D. N. & Khajavikhan, M. Parity-time-symmetric microring lasers. *Science* **346**, 975–978 (2014).
