# Supplementary material for: Complex skin modes in non-Hermitian coupled laser arrays
Source: Light Sci Appl. 2022 Nov 28;11:336. doi: 10.1038/s41377-022-01030-0 (PMC9705320; doi:10.1038/s41377-022-01030-0)
Supplement: Supplementary file 4 — Editor Note and Peer Review Reports [file 41377_2022_1030_MOESM4_ESM.pdf]

Editor Note for

Complex Skin Modes in Non-Hermitian Coupled Laser Arrays

Dear readers:

We have decided to publish this manuscript, Complex Skin Modes in Non-Hermitian Coupled Laser Arrays, after the peer reviews. This paper has received mixed comments from five dedicated reviewers. These comments were either strongly positive or strongly negative. After these inputs were carefully analyzed, we think it would be the best for the interest of the authors, reviewers, and readers to have this manuscript published even there are negative comments. We also publish the negative comments, together with the authors replies. LSA is willing to consider potentially high impact paper which may not be a perfect paper during the review process. We wish this publication will stimulate fruitful scientific discussions and experimental confirmations.

Xi-Cheng Zhang

Editor-in-chief of Light: Science & Applications

Peer Review Report for

Complex Skin Modes in Non-Hermitian Coupled Laser Arrays

Comments from Reviewer #2 -1st Round

The manuscript by Liu and co-authors titled "Complex Skin Modes in Non-Hermitian Coupled Laser Arrays" describes an experimental demonstration of the Hatano-Nelson model in coupled laser arrays. Microring resonators are connected by waveguides on III-V active platform. The coupling between resonators is controlled by the amplification and phase accumulation on the waveguide and can be made asymmetric (e.g., right coupling being stronger than left coupling) to mimic the Hamiltonian of Hatano-Nelson model. Because opposite energy circulations (clockwise (CW) versus counter-clockwise (CCW)) in the ring resonators experience opposite asymmetry in the coupling strengths, the authors introduce unidirectional (CW to CCW but not vice versa) coupling to promote CCW power circulation. Experimentally the authors first show single-mode lasing in a 2-element and a 5-element array, both with asymmetric intensity distribution (skin effect). Then multi-mode laser arrays are also shown with skin effects. The authors claim that the asymmetric intensity distribution matches the skin-effect from Hatano-Nelson model quantitatively and that the single-mode and multi-mode lasing operations are expected from link length satisfying $\beta L \cong m\pi$ and (m+1/2)π respectively.

The topic under study is interesting to the community, however, there is a severe disconnection between the authors' theory and the experimental results. The authors claim that the g-factor characterizing the asymmetry in the coupling is ± 3.33 in Fig.2 and ± 3.47 in Fig. 3. With the definition supplied by the authors (g=1/2ln($\gamma l/\gamma u$)), the Hamiltonian is {{0, i*e^(3.33)},{i*e^(-3.33),0}. The eigenvector of this Hamiltonian feature amplitude distribution of 27.9:1 which is 780:1 in intensity. This does not match with the observed intensity distribution (nor the theory result) in Fig. 2b and 2c. The same problem happens to Fig. 3 c,f, and i as well, where the intensity distribution should be {1.00, 0.0029, 3.8E-6, 2.7E-9, 8,8E-13} among the 5 rings according to g=3.47, much more concentrated on the edge than the authors show. Possibly there is an error in the g factor or in the authors' theoretical calculation. But without any description of how the g factor is determined by the authors, the quantitate match that the authors claim is rather unconvincing.

More critically, there is a fundamental flaw in the authors' theory about the phase of the coupling coefficient, which is a critical piece in the explanation of single-mode versus multi-mode operation. The authors design the waveguides to be ~95 μm. For such long waveguides, the phase accumulation βL is very sensitive to the wavelength. In Fig. 4e and 4i, we can observe >2 nm separation in wavelength between different modes. Taking into the fact that with a longer wavelength, the effective mode index also decreases, a simple simulation shows that the variation in the accumulation phase βL due to wavelength variation is (2π*n_1/λ_1 - 2π*n_2/λ_2)*L =~ (2π*2.2784/1.592μm-2π*2.2759/1.594μm)*95μm > 0.5π. Hence, even if the phase condition βL~(m+1/2)π is met for one of the lasing peaks, it cannot be met for the other lasing peak that is 2nm from it.

In general, in the manuscript, there are quite some unconvincing claims, especially around the phase of the

coupling terms. For long waveguides shown here, it is hard to believe that the coupling phase is controlled the way the authors claim, and in my opinion, it is erroneous to claim that such a phase condition holds for a wavelength range across several nanometers. I believe the manuscript is not suitable for publication unless the authors can fix the disconnection between the claimed Hatano-Nelson model and their experimental system. My other comments are listed below which may help the authors fix this disconnection.

1. The authors claim that "This coupling scheme affects the spontaneous emission by modifying the density of states, thus promoting an energy circulation in the rings only in one direction". Without doubt, this (unidirectional) coupling scheme affects the lasing emission by modifying the eigenmode, but I believe the spontaneous emission is not modified by the EP in the claimed way. The spontaneous emission may very well couple to the other energy circulation direction as proved by [H.-Z. Chen, et al., Revealing the Missing Dimension at an Exceptional Point, Nat. Phys. 1 (2020).]. The claim made by the authors here about spontaneous emission is very misleading, and it seems unrelated to the lasing operation discussed in the manuscript.

2. Regarding Fig. 2, the authors claim that "indeed the energy circulates in a counter-clockwise direction in the rings". However, the energy in clockwise (CW) is nonzero in Fig. 2b and 2c. Moreover, in Fig. 2c, for the right ring, the intensity in CW is similar (if not stronger) compared to the CCW direction. In Fig. 3h, ring 5, we also see that the CW mode is stronger than the CCW mode, in direct disagreement with the authors' models. This is another disconnection between the authors' model and the experimental result.

3. Regarding the optical pumping region, when the lower arms are pumped, the region is always smaller than when the upper arms are pumped, and the region does not cover the whole waveguide. What would be the reason for such asymmetry between upper and lower arms? What would happen if the whole lower arms were pumped?

4. What is the material gain under pumping and the intrinsic loss when not under pumping for the waveguides? Do they match the g factor provided by the authors? Also, the carrier density would impact the effective index in the waveguides, do they affect the designed coupling phase βL, or can it be used to control βL? For example, in Fig. S5 in supplementary material, can the authors justify that with varying pumping regions, the coupling phase stays a constant?

5. The authors claim that Fig. 2 indicates that "the proper coupling phase conditions are experimentally established" because the spectra show single peak. It is unconvincing because the splitting in frequency between coupled modes is proportional to sqrt($\gamma\_l*\gamma\_u$) and can be below the linewidth of the laser. The authors should provide the absolute value of $\gamma\_l*\gamma\_u$ for this to be more convincing, instead of only providing the factor g. Also since the authors have 16 varying lengths in the design, the phase condition should be established by measuring designs with varying lengths instead of the characterization of a single device, if the phase condition is indeed controlled by the design.

6. The discussion about real vs imaginary eigen-energies (around Fig. 1c) seems disconnected from the body of the manuscript, as the majority of the coupling coefficients discussed later are not real-valued.

Responses from authors -1st Round

The manuscript by Liu and co-authors titled "Complex Skin Modes in Non-Hermitian Coupled Laser Arrays" describes an experimental demonstration of the Hatano-Nelson model in coupled laser arrays. Microring resonators are connected by waveguides on III-V active platform. The coupling between resonators is controlled by the amplification and phase accumulation on the waveguide and can be made asymmetric (e.g., right coupling being stronger than left coupling) to mimic the Hamiltonian of Hatano-Nelson model. Because opposite energy circulations (clockwise (CW) versus counter-clockwise (CCW)) in the ring resonators experience opposite asymmetry in the coupling strengths, the authors introduce unidirectional (CW to CCW but not vice versa) coupling to promote CCW power circulation. Experimentally the authors first show single-mode lasing in a 2-element and a 5-element array, both with asymmetric intensity distribution (skin effect). Then multi-mode laser arrays are also shown with skin effects. The authors claim that the asymmetric intensity distribution matches the skin-effect from Hatano-Nelson model quantitatively and that the single-mode and multi-mode lasing operations are expected from link length satisfying $\beta L \cong m\pi$ and (m+1/2)π respectively.

The topic under study is interesting to the community, however, there is a severe disconnection between the authors' theory and the experimental results. The authors claim that the g-factor characterizing the asymmetry in the coupling is ± 3.33 in Fig.2 and ± 3.47 in Fig. 3. With the definition supplied by the authors (g=1/2ln($\gamma l/\gamma u$)), the Hamiltonian is {{0, i*e^(3.33)},{i*e^(-3.33),0}. The eigenvector of this Hamiltonian feature amplitude distribution of 27.9:1 which is 780:1 in intensity. This does not match with the observed intensity distribution (nor the theory result) in Fig. 2b and 2c. The same problem happens to Fig. 3 c,f, and i as well, where the intensity distribution should be {1.00, 0.0029, 3.8E-6, 2.7E-9, 8,8E-13} among the 5 rings according to g=3.47, much more concentrated on the edge than the authors show. Possibly there is an error in the g factor or in the authors' theoretical calculation. But without any description of how the g factor is determined by the authors, the quantitate match that the authors claim is rather unconvincing.

We would like to thank the reviewer for pointing out our calculation mistake. For Fig.2, the ratio between the gain of the upper and lower links is 27.9 and the g factor should be calculated as $g = (1/2)\ln(\gamma_l/\gamma_u) = 1.66$. Similarly, for Fig. 3, the ratio between the gain of the upper and lower links is 32 and the g factor should be calculated as $g = (1/2)\ln(\gamma_l/\gamma_u) = 1.73$. We missed the factor ½ in our calculations. It is now corrected.

The g factors are determined by the total amplification/attenuation of the links. The material gain and intrinsic loss were previously reported (please see for example Science 346, 975 (2014) and its supplementary file). At the pump levels reported in the manuscript, the waveguide gain is $\gamma \cong 50\ \mathrm{cm}^{-1}$ and the intrinsic loss is $\alpha \cong -450\mathrm{cm}^{-1}$. The upper link is fully pumped, while the lower link is only partially left unpumped $l_1 \cong 30\ \mu\mathrm{m}$ (the pumped part), $l_2 \cong 65\mu\mathrm{m}$ (the unpumped region) (please see Fig. R1 for the pump profile). The gain/loss of the upper and lower links are therefore $\gamma_u = e^{\gamma(95\mu\mathrm{m})} = 1.61$ and $\gamma_l = e^{\gamma(30\mu\mathrm{m})+\alpha(65\mu\mathrm{m})} = 0.06$, respectively. This gives a g factor that is $g = (1/2)\ln(\gamma_l/\gamma_u) \cong 1.62$- which is quite close to what is deduced from the measurement.

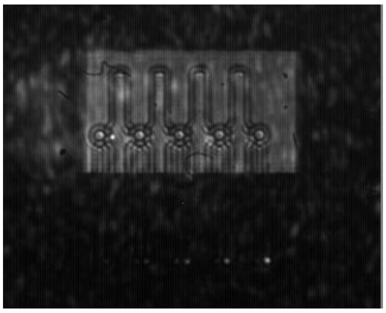

Figure R1. The pump profile during the experiments.

The g factors corresponding to these two experimental results (they were off by a factor of 2) are now corrected in

Page 4, paragraph 3 lines 5&6 and Page 6, paragraph 1 lines 4 in the revised manuscript.

More critically, there is a fundamental flaw in the authors' theory about the phase of the coupling coefficient, which is a critical piece in the explanation of single-mode versus multi-mode operation. The authors design the waveguides to be ~95 μm. For such long waveguides, the phase accumulation βL is very sensitive to the wavelength. In Fig. 4e and 4i, we can observe >2 nm separation in wavelength between different modes. Taking into the fact that with a longer wavelength, the effective mode index also decreases, a simple simulation shows that the variation in the accumulation phase βL due to wavelength variation is (2π*n_1/λ_1 - 2π*n_2/λ_2)*L =~ (2π*2.2784/1.592μm-2π*2.2759/1.594μm)*95μm > 0.5π. Hence, even if the phase condition βL~(m+1/2)π is met for one of the lasing peaks, it cannot be met for the other lasing peak that is 2nm from it.

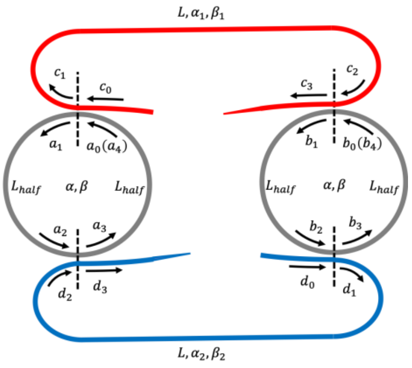

Figure R1. The geometry of the problem.

There is no fundamental problem with our theory. Perhaps, part of the confusion arises from the simple analysis provided by the reviewer in his/her critique, given that it does not fully capture the physics of the arrangement used. We would like to draw the reviewer's attention to the fact that here the frequency splitting is governed by both strength and phase of the coupling. In this respect, resonance frequencies are already adjusted to account for the phase (determined by the lengths of the links) of the coupling regions.

In order to properly account for the phase variation in the links, one needs to incorporate that in the model and calculate the resonant frequency and magnitude of the modes accordingly. Here we provide a detailed analysis for the case of two resonators. We formulate the problem based on spatial coupled mode theory that relates the fields a_0 and b_0 to the fields at the same locations after one round trip in the cavity labeled as a_4 and b_4, respectively (please see Fig. R2).

The resulting transfer matrix is expressed as follows:

$$\begin{bmatrix} a_4 \\ b_4 \end{bmatrix} = e^{2(j\beta+\alpha)L_{half}} \begin{bmatrix} \sigma^2 + \kappa^4 e^{(j\beta_1+\alpha_1)L} e^{(j\beta_2+\alpha_2)L} & \kappa^2 \sigma e^{(j\beta_2+\alpha_2)L} \\ \kappa^2 \sigma e^{(j\beta_1+\alpha_1)L} & \sigma^2 \end{bmatrix} \begin{bmatrix} a_0 \\ b_0 \end{bmatrix} \quad \text{(Eq. R1)}$$

Where σ and κ are the through and cross coupling between the ring and link, $\alpha = \alpha_1$is the gain of the fully pumped link as well as the ring resonator, and α_2 is the effective gain/loss of the other link. Here, β, β_1 and β_2 are the propagation constants of the resonators, upper link, and effective propagation constant of the lower link, and L is the length of the links and $L_{half} = \pi R$, where R is the radius of the ring resonators.

The eigenvalues of the system are given by:

$$\lambda_{1,2} = \frac{1}{2} e^{2(j\beta+\alpha)L_{half}} \left(\begin{matrix} \kappa^4 e^{(\alpha_1+\alpha_2+j\beta_1+j\beta_2)L} + 2\sigma^2 \\ \pm\sqrt{\kappa^8 e^{2(\alpha_1+\alpha_2+j\beta_1+j\beta_2)L} + 4\kappa^4\sigma^2 e^{(\alpha_1+\alpha_2+j\beta_1+j\beta_2)L}} \end{matrix} \right) \quad \text{(Eq. R2)}$$

Similarly, the corresponding eigenvectors are as follows:

$$V_{1,2} = \begin{bmatrix} \frac{e^{-(j\beta_1+\alpha_1)L}}{2\kappa^2\sigma} \left(\begin{matrix} \kappa^4 e^{(\alpha_1+\alpha_2+j\beta_1+j\beta_2)L} \\ \pm\sqrt{\kappa^8 e^{2(\alpha_1+\alpha_2+j\beta_1+j\beta_2)L} + 4\kappa^4\sigma^2 e^{(\alpha_1+\alpha_2+j\beta_1+j\beta_2)L}} \end{matrix} \right) & 1 \end{bmatrix}^T \quad \text{(Eq. R3)}$$

In order to find the resonance frequency of the modes, one must find the wavelength at which the phase of the eigenvalues becomes zero. The magnitude of the eigenvalue gives the gain/loss of the mode.

Below we provide the plots associated with the phase and magnitude of the eigenvalues for two values of L (specifically when $L = 95.065\ \mu\text{m}$ and the other case is $L = 94.885\ \mu\text{m}$) and as a function of wavelength. Given that $n_{\text{eff}} = 2.24$, at a wavelength of $\lambda = 1.595\ \mu\text{m}$, this length difference results in $\sim\pi/2$ phase shift.

As the reviewer can see, at $L = 94.885\ \mu\text{m}$ which corresponds to a phase condition of $m\pi + \pi/2$, two modes appear that satisfy the resonant condition while being $\cong 1\ \text{nm}$ apart, both having a very similar magnitude- therefore they are both lasing.

On the other hand, when $L = 95.065\ \mu\text{m}$, corresponding to a $m\pi$ phase condition, the two modes are lasing at the same wavelength, with varying magnitude of the eigenvalues (one considerably higher than the other one). Since the modes are at the same frequency, the one with the higher magnitude of eigenvalue is going to lase, thus leading to single mode lasing.
The analysis can be readily expanded to a 5-ring arrangement. However, the above analysis should have already clarified the point for the reviewer as how the phase condition is satisfied, without delving into the complexity associated with the 5-ring system.
To

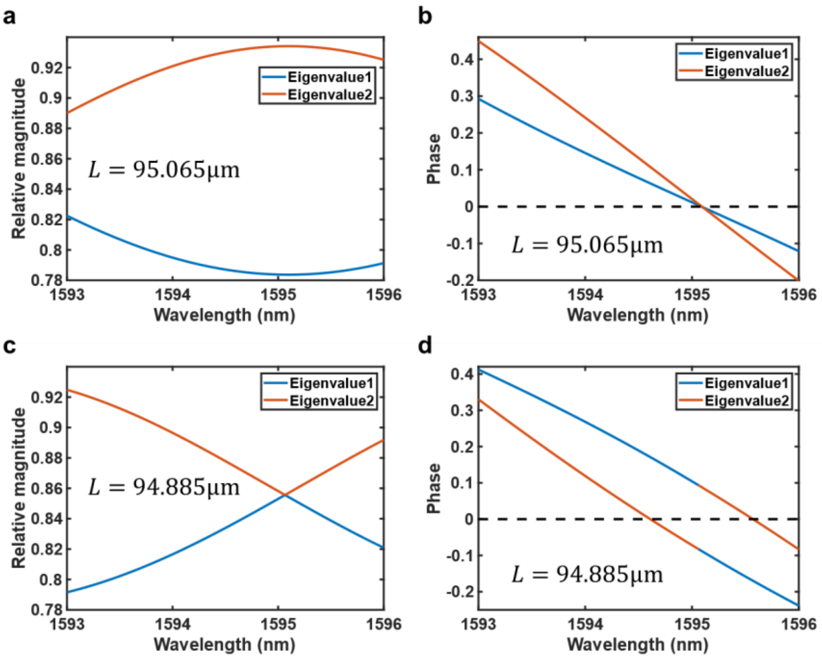

Figure R2. Relative magnitude and phase of the eigenvalues for two different link lengths apart by $\pi/2$ phase shift. For (a) and (b), $L = 95.065\ \mu m$. For (c) and (d), $L = 94.885\ \mu m$.

make this aspect more clear, we have now added this analysis to the Supplementary Section 7. Please see the revised Supplementary Information, Page 9.
In general, in the manuscript, there are quite some unconvincing claims, especially around the phase of the coupling terms. For long waveguides shown here, it is hard to believe that the coupling phase is controlled the way the authors claim, and in my opinion, it is erroneous to claim that such a phase condition holds for a wavelength range across several nanometers. I believe the manuscript is not suitable for publication unless the authors can fix the disconnection between the claimed Hatano-Nelson model and their experimental system. My other comments are listed below which may help the authors fix this disconnection.

The detailed explanation to the previous question should address the comment about the phase of the coupling terms. If the main reason for the recommendation made was based on this comment, we hope at this point the reviewer reconsider his/her decision. We would also like to remind the reviewer that the experimental results fully confirm the Hatano-Nelson model that indicates, regardless of the phase, the energy distribution is tilted towards one side of the array depending on the ratio of $|\kappa_R/\kappa_L|$. The phase merely changes the number of modes lasing in the system. Below we address the other comments made by the reviewer in the order they appear.

1. The authors claim that "This coupling scheme affects the spontaneous emission by modifying the density of states, thus promoting an energy circulation in the rings only in one direction". Without doubt, this (unidirectional) coupling scheme affects the lasing emission by modifying the eigenmode, but I believe the spontaneous emission is not modified by the EP in the claimed way. The spontaneous emission may very well couple to the other energy circulation direction as proved by [H.-Z. Chen, et al., Revealing the Missing Dimension at an Exceptional Point, Nat. Phys. 1 (2020).]. The claim made by the authors here about spontaneous emission is very misleading, and it seems unrelated to the lasing operation discussed in the manuscript.

We would like to point out that the claim we made in the manuscript is valid in the context of laser arrangements, while the reference provided by the reviewer investigates an altogether different system. We would also like to refer the reviewer to the extensive discussion provided in the supplementary of Nat. Phys. 17, 704 (2021).

In the paper brought up by the reviewer, the emission occurs from a specific localized emitter that is properly placed to match the required chirality reversal conditions as described in their Eq. (2). In that paper, the authors have included an extensive discussion in their Supplementary Information (Section 4), demonstrating a case where emission occurs from the entire cavity and therefore the chirality of the emitted wave is not reversed. In this respect, the authors of the referenced 2020 Nature Physics article point out the following (paragraph 2, page 3)

"*We note that the chirality-reversal phenomenon is in stark contrast to exceptional point lasers, where the properties extracted are solely determined by the chiral eigenstates, and not the Jordan vectors. When the emitter position deviates from φ0 = π/4l, the interference condition will change accordingly. In exceptional point lasers, the emitters are distributed over the entire cavity, and therefore the chirality of the lasing mode is determined by the integrated radiation of all emitters. On the basis of the steady-state ab initio laser theory (SALT)35,36, we find that a uniform gain profile leads to lasing in the coalesced eigenstate, but not in the Jordan vector*"

The fact that the spatial and spectral cavity modes affect the spontaneous emission due to Purcell effect is well established. One may question the extent of it, but not its presence.

To clarify this, we have now referred to our paper in Nature physics in the revised manuscript, Page 3, Paragraph 2, Line 8.

2. Regarding Fig. 2, the authors claim that "indeed the energy circulates in a counter-clockwise direction in the rings". However, the energy in clockwise (CW) is nonzero in Fig. 2b and 2c. Moreover, in Fig. 2c, for the right ring, the intensity in CW is similar (if not stronger) compared to the CCW direction. In Fig. 3h, ring 5, we also see that the CW mode is stronger than the CCW mode, in direct disagreement with the authors' models. This is another disconnection between the authors' model and the experimental result.

The residual small power that one sees in the other waveguide is due to partial pumping of the bus waveguides in order to reduce the loss in the path towards the gratings. We suggest that the reviewer compares the results of Fig. 3h to those in Figs. 3b and e, or Fig.2b with that of Fig. 2c to see that it is generally the trend.

Nevertheless, unidirectionality does not mean that the power in one direction is absolutely zero, there is always a small amount of spontaneous emission power that exists in the other direction. In our previous works, for example in [Opt. Express 26, 27153-27160 (2018)] we measured the extinction ratio (that is clearly not infinity).

To further clarify this aspect, we have added a sentence in the manuscript. Please see Page 5, Paragraph 1, Line 5 in the revised manuscript.

3. Regarding the optical pumping region, when the lower arms are pumped, the region is always smaller than when the upper arms are pumped, and the region does not cover the whole waveguide. What would be the reason for such asymmetry between upper and lower arms? What would happen if the whole lower arms were pumped?

Pumping the entire lower links also pumps the majority of the bus waveguide because of the way the structure is fabricated and the way we use optical pumping. Given the fact that the bus waveguide is also made of active III-V material, pumping creates a strong background signal at the output gratings from spontaneous emission, thus reducing the visibility of the extinction ratio between the intensity profiles from the two directions. This background can smear the true state of light in the rings- that is why we avoid fully pumping the bus waveguides and hence the lower links.

To clarify this point, we have added a sentence in the manuscript. Please see Page 5, Paragraph 1, Line 7 in the revised manuscript.

4. What is the material gain under pumping and the intrinsic loss when not under pumping for the waveguides? Do they match the g factor provided by the authors? Also, the carrier density would impact the effective index in the waveguides, do they affect the designed coupling phase βL, or can it be used to control βL? For example, in Fig. S5 in supplementary material, can the authors justify that with varying pumping regions, the coupling phase stays a constant?

The waveguide gain under pumping varies, and for the value of pump reported here is $\cong 50\ \mathrm{cm}^{-1}$ (please see our other works, for example, Science 346, 975 (2014) and its supplementary file). The loss also varies based on the level of pumping and background light and here it should be around $\cong -450\ \mathrm{cm}^{-1}$. These values match remarkably well with our experimental results.

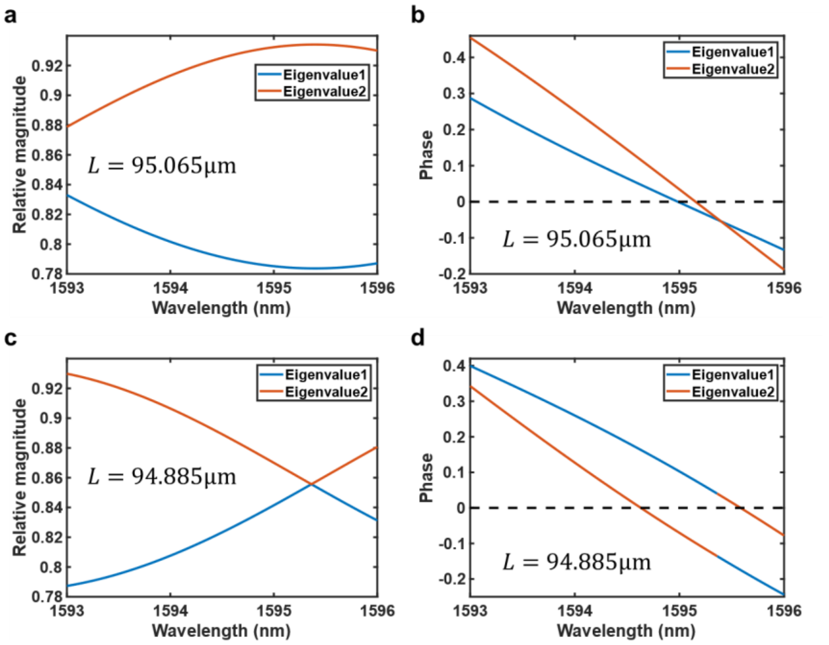

Figure R3. Relative magnitude and phase of the eigenvalues for two different link lengths apart by $\pi/2$ phase shift with gain induced phase change. For (a) and (b), $L = 95.065\ \mu m$. For (c) and (d), $L = 94.885\ \mu m$.

The change in the phase due to excess carrier density follows $\Delta\phi = \frac{\alpha}{2}\Delta g L$. For $\alpha = 2$, the resulting phase from the fully pumped link is $\Delta\phi = 0.5$ rad and for the partially pumped link it is $\Delta\phi = 0.15$ rad. This results in a net change of $\Delta\phi = 0.35$ rad or roughly $\pi/10$ phase difference. This phase difference minimally affects the performance of the system. The eigenvalues as calculated above (please see Eq. R2) with this extra phase are given in Fig. R4.

As it can be seen, for the case $m\pi$ phase condition, the system now supports two modes with very small frequency separation, but one of the eigenvalues has a considerably smaller magnitude. For the case of $m\pi + \pi/2$, no discernable difference is observed. We repeated the simulation for $\alpha = 3$, and the result was similar.

We have now added this analysis in the Supplementary Section 8. Please see the revised Supplementary Information, Page 11.

5. The authors claim that Fig. 2 indicates that "the proper coupling phase conditions are experimentally established" because the spectra show single peak. It is unconvincing because the splitting in frequency between coupled modes is proportional to sqrt(γ_l*γ_u) and can be below the linewidth of the laser. The authors should provide the absolute value of γ_l*γ_u for this to be more convincing, instead of only providing the factor g. Also since the authors have 16 varying lengths in the design, the phase condition should be established by measuring designs with varying lengths instead of the characterization of a single device, if the phase condition is indeed controlled by the design.

We refer the reviewer to the analysis with the eigenvalues and eigenvectors (Eq. R3). As the reviewer can see the splitting in frequency is more involved than sqrt(γ_l*γ_u).

As for the measurements, we indeed characterized 16 patterns with varying lengths. The results provided in the manuscript show the ultimate cases of when the two frequencies are furthest apart and when single mode lasing is attained.

To clarify this point, we have added a sentence in the manuscript. Please see Page 4, Paragraph 3, Line 2 in the revised manuscript.

6. The discussion about real vs imaginary eigen-energies (around Fig. 1c) seems disconnected from the body of the manuscript, as the majority of the coupling coefficients discussed later are not real-valued.

The discussion around Fig. 1c serves as an introduction to the Hatano-Nelson model under periodic boundary condition- that was in fact their original model. With all due respect to the reviewer, we would like to retain Fig. 1c for completeness.

We would like to thank again the referee for the time he/she spent in reviewing our manuscript. We hope the detailed response above now clarifies the main points of the paper and the reviewer finds our work suitable for publication in Nature LSA.

Comments from Reviewer #2 -2nd Round

The authors have not addressed the fundamental problem regarding the phase of the coupling coefficient in the long waveguides. It is especially problematic in their explanation of multimode devices. Dispersion in these long waveguides would make the propagation phase super sensitive to small wavelength changes, and it is totally unreasonable to assume a constant propagation phase in multimode devices with a wavelength span of more than a couple of nanometers. In the rebuttal, the authors provided a quantitative model that explains a 2-ring array with a wavelength span of ~1 nm and claims that such a model can be readily expanded to a 5-ring arrangement. This claim is simply wrong, because in a 5-ring array the wavelength span won't be as small as 1nm and the model would fail to agree with their story produced by assuming βL is a constant. If we estimate the Δβ*L caused by a 1 nm wavelength span, it is actually ~0.3 π and is probably about the upper limit before the simple model fails. It is unreasonable to claim such a model would be readily expanded to a larger array, as the wavelength span increases with array size due to coupling.

My comment is very easy to understand. I suggest we simply calculate the FSR for a waveguide as long as 95μm (the minimum wavelength change that causes Δβ*L = 2π), which is Δλ=λ^2/(n_group*L)=1550nm^2/(4*95μm)=6.3 nm, meaning a wavelength change of 6.3 nm would cause the propagation phase to change 2π. In the multimode array shown in Fig. 4, the total wavelength span in the spectra is more than 2nm, which means the propagation phase varies more than 0.5 π. Within the 2nm span, for some wavelengths, it would have β*L = mπ and for some other ones, they would have β*L = mπ+0.5pi. The authors basically are claiming that for all the wavelengths across the 2nm range the propagation phase is β*L = mπ. This mistake is obvious.

I would suggest the authors to actually extend their model (Eq. R1) to a 5-ring array if they still think they can find a 5-ring array with multimode lasing when $\beta L= m\pi$. I think the authors would inevitably find that within the wavelength span they also have a certain wavelength that satisfies $\beta L= m\pi + 0.5\pi$ and it would be impossible to have all the eigenvalues around the same magnitude. Also, the authors should not make the mistake as they did in this rebuttal to use a constant effective index of 2.24 when they should use a group index of ~4.

Because the multimode devices and the claims around $\beta L = m\pi$ make up a large part of the manuscript and because the mistake is so obvious, I have to hold my recommendation of rejection.

Responses from authors -2nd Round

The authors have not addressed the fundamental problem regarding the phase of the coupling coefficient in the long waveguides. It is especially problematic in their explanation of multimode devices. Dispersion in these long waveguides would make the propagation phase super sensitive to small wavelength changes, and it is totally unreasonable to assume a constant propagation phase in multimode devices with a wavelength span of more than a couple of nanometers. In the rebuttal, the authors provided a quantitative model that explains a 2-ring array with a wavelength span of ~1 nm and claims that such a model can be readily expanded to a 5-ring arrangement. This claim is simply wrong, because in a 5-ring array the wavelength span won't be as small as 1nm and the model would fail to agree with their story produced by assuming βL is a constant. If we estimate the $\Delta\beta$*L caused by a 1 nm wavelength span, it is actually ~0.3 π and is probably about the upper limit before the simple model fails. It is unreasonable to claim such a model would be readily expanded to a larger array, as the wavelength span increases with array size due to coupling.

My comment is very easy to understand. I suggest we simply calculate the FSR for a waveguide as long as 95µm (the minimum wavelength change that causes $\Delta\beta$*L = 2π), which is $\Delta\lambda$ = λ^2/(n_group*L) = 1550nm^2/(4*95µm) = 6.3 nm, meaning a wavelength change of 6.3 nm would cause the propagation phase to change 2π. In the multimode array shown in Fig. 4, the total wavelength span in the spectra is more than 2nm, which means the propagation phase varies more than 0.5 π. Within the 2nm span, for some wavelengths, it would have β*L = mπ and for some other ones, they would have β*L = mπ+0.5pi. The authors basically are claiming that for all the wavelengths across the 2nm range the propagation phase is β*L = mπ. This mistake is obvious.

I would suggest the authors to actually extend their model (Eq. R1) to a 5-ring array if they still think they can find a 5-ring array with multimode lasing when $\beta L= m\pi$. I think the authors would inevitably find that within the wavelength span they also have a certain wavelength that satisfies $\beta L= m\pi + 0.5\pi$ and it would be impossible to have all the eigenvalues around the same magnitude. Also, the authors should not make the mistake as they did in this rebuttal to use a constant effective index of 2.24 when they should use a group index of ~4.

We would like to thank again the reviewer for his/her time. We would also like to point out that, FSR is a concept that is only meaningful in the context of resonators (not waveguides as the reviewer stated). We believe the comment made by the reviewer about the group index (4) to be used instead of effective index (2.24) is perhaps also motivated by the fact that the reviewer is treating the waveguides as resonators.

We also would like to clarify that the model provided for 2-element system (Supplementary Section 7) is indeed a theoretical analysis- not a quantitative one. As per reviewer request, here we provide a detailed analysis for the 5-element case to show that the reviewer's simple analysis does not apply in this case as well.

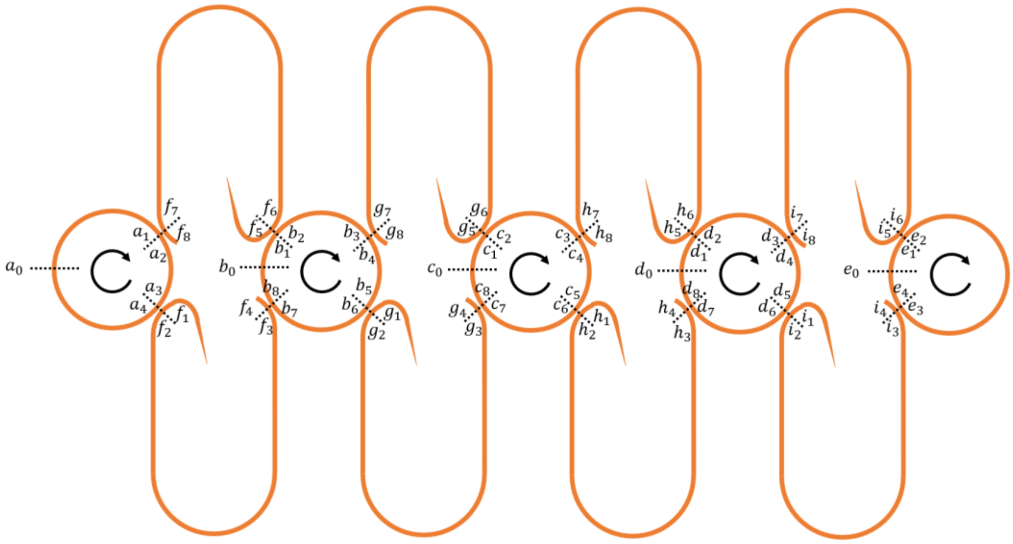

Figure RR1. 5-element microring laser array that supports multimode operations. Microring lasers are assumed to support clockwise mode only.

Here, we use the spatial coupled mode theory and monitor the field amplitudes in various locations of the structure (Fig. RR1). The transfer matrix of the system can be written as:

$$\begin{bmatrix} A_{11} & A_{12} & 0 & 0 & 0 \\ A_{21} & A_{22} & A_{23} & 0 & 0 \\ 0 & A_{32} & A_{33} & A_{34} & 0 \\ 0 & 0 & A_{43} & A_{44} & A_{45} \\ 0 & 0 & 0 & A_{54} & A_{55} \end{bmatrix} \begin{bmatrix} a_0 \\ b_0 \\ c_0 \\ d_0 \\ e_0 \end{bmatrix} = \hat{A} \begin{bmatrix} a_0 \\ b_0 \\ c_0 \\ d_0 \\ e_0 \end{bmatrix} = \Lambda \begin{bmatrix} a_0 \\ b_0 \\ c_0 \\ d_0 \\ e_0 \end{bmatrix} \qquad \text{(RR1)}$$

where Λ is the system's eigenvalue and $[a_0 \quad b_0 \quad c_0 \quad d_0 \quad e_0]^T$ is the eigenvector. $A_{mn}{}'s$ in the matrix $\hat{A}$ are the coupling coefficients between elements (please see the Supplementary Section 9 for the exact derivation). Even though the eigenvalues can be analytically expressed (using for example Mathematica), to avoid writing pages of formula, here we only focus on a numerical solution. Below (in Fig. RR2) we provide the plots associated with the phase and magnitude of the eigenvalues as a function of wavelength for two values of L (specifically when $L = 95.065\ \mu m$ (resulting in $\beta L \cong m\pi$) and the other case is $L = 94.885\ \mu m$ (where $\beta L \cong (m + 0.5)\pi$)).

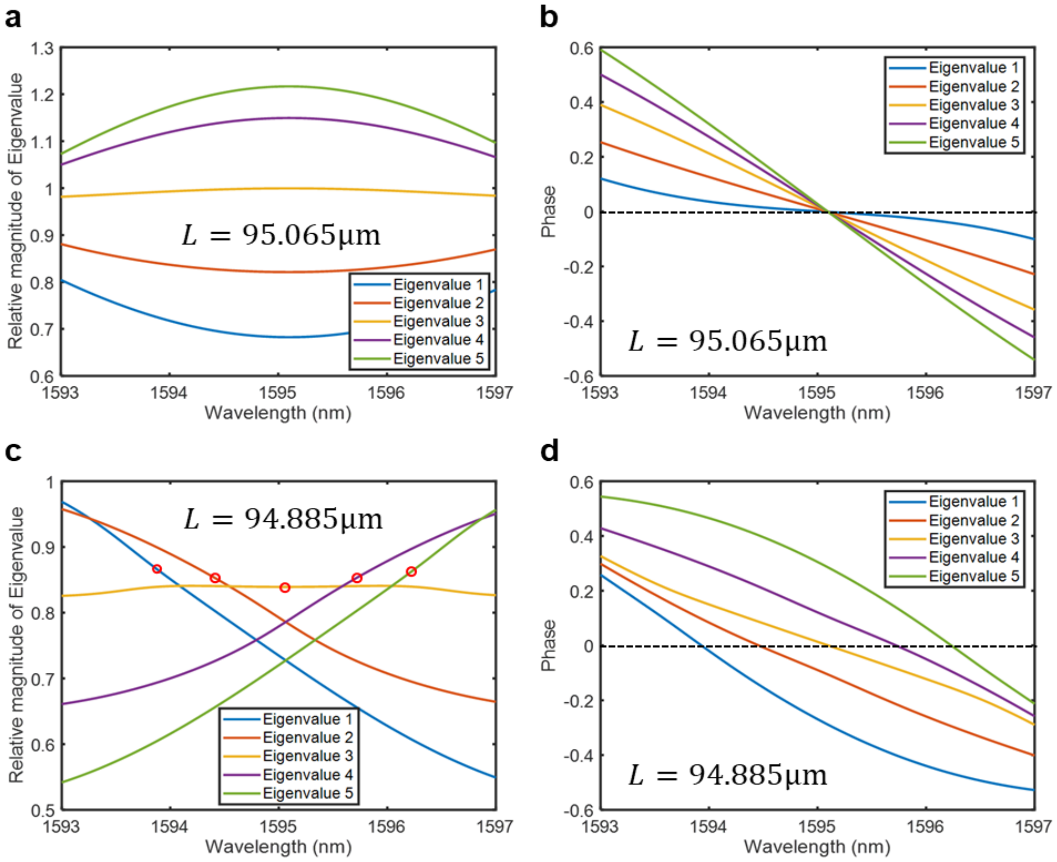

Figure RR2. Relative magnitude and phase of the eigenvalues for two different link lengths featuring $\pi/2$ phase shift. For (a) and (b), $L = 95.065\ \mu m$. For (c) and (d), $L = 94.885\ \mu m$. Red circles in (c) indicate the resonance wavelengths of individual modes.

As the reviewer can see, when $L = 95.065\ \mu m$, corresponding to a $m\pi$ phase condition, the five modes are all lasing at the same wavelength, with varying magnitude of the eigenvalues. Since the modes are at the same frequency, only the one with the largest eigenvalue will lase, thus leading to a single mode lasing operation. On the other hand, when $L = 94.885\ \mu m$, which corresponds to a phase condition of $m\pi + \pi/2$, five modes appear that satisfy the resonant condition while being $\sim 2\ nm$ apart (marked by red circles in Fig. RR2(c)), thus leading to multimode lasing. Since the splitting between individual modes is less than the resolution of our spectrometer (0.64 nm), the multi-mode spectra shown in the manuscript does not resolve all the modes.

One should notice that, in Fig. RR2 (b), 5 modes meet the condition of $\beta L \cong m\pi$ at ~1595 nm. While in Fig.RR2 (d), only one of the modes (mode 3) meets the condition of $\beta_3 L \cong m\pi + \pi/2$, while the lasing wavelengths of modes 2 and 4 satisfy $\beta_{2,4} L \cong \beta_3 L \pm \pi/4$, and modes 1 and 5 result in $\beta_{1,5} L \cong \beta_3 L \pm \pi/2$. These deviations are indeed what causes the system to become multimoded.

Again, we would like to reiterate that the simple model that the reviewer uses to justify his/her claim does not accurately describe this system. It seems to us that the reviewer uses temporal coupled mode theory to describe the operation of the rings and spatial coupled mode theory to justify the role of the links- instead of either using fully spatial or fully temporal coupled mode analysis to describe the entire system.

For the purpose of clarifying this point, we have added the above detailed analysis for the 5-element system as Supplementary Section 9. Please see the revised Supplementary Information, Page 12.

Because the multimode devices and the claims around $\beta L = m\pi$ make up a large part of the manuscript and because the mistake is so obvious, I have to hold my recommendation of rejection.

Given that we have now analytically and numerically shown that our claims about the multimode response are indeed fully valid not only for the case of 2-element system, but also for the 5-element array, we would like to kindly ask the reviewer to reconsider his/her decision.

Comments from Reviewer #2 -3rd Round

The concept of FSR is, for sure, not limited to resonators. One example is FSR in Mach-Zehnder interferometers. See, for example, [Chrostowski and Hochberg, Silicon photonics design: from devices to systems, Cambridge University Press, 2015] Equation (4.20). But really there is no need to debate about this, since I explicitly defined what I meant by FSR in my previous comment. Note that group index is used in Equation (4.20) of [Chrostowski and Hochberg], for the same reason that I suggested, to capture the dispersion of the waveguide. When calculating the dependence of βL on wavelength, group index should be used, instead of assuming a constant effective index which ignores the dispersion. Albeit my repeated suggestions, the authors still ignore the waveguide dispersion in their model, which results in likely a factor of ~1.7 error in their $\Delta\beta L$ vs wavelength, which is significant since the waveguide is very long, with βL~266π. If the authors want to claim quantitative agreement between their model and experiment, this dispersion must be considered. It actually raises one's doubt about the authors' result when they claim such high level of agreement between experiment and theory when a critical factor, waveguide dispersion, is completely ignored. The authors should use a mode solver to calculate the effective index for each wavelength (or simply, as I suggested, calculate the group index) in their structure and modify their model, if they want to claim quantitative agreement in a system with a waveguide as long as 133x wavelength. This is obviously critical for such a long waveguide and can significantly change the authors' estimation of coupling coefficients.

About the detailed model that authors provided for the 5-element array, this is indeed progress towards the right direction. However, I find the argument and evidence laid out by the authors still not sufficiently convincing and far from considering publication on Light.

First of all, since the authors have repeatedly stated that my model is simple and does not accurately describe the system. Let me remind the authors that their original model, which is now still the model in the main text, is even simpler, and does not accurately describe the system either. Now that the authors accept that out of the 5 modes in Fig. RRR2 (B), only one mode satisfies βL= mπ+π/2, while two of the modes satisfy βL=mπ, it seems sufficient to say that the claims (for example around Fig. 4) about the link length satisfying βL -> mπ + π/2 is oversimplifying and confusing, if not inaccurate.

Moreover, there is still a significant disconnection between the theory and the experiment around the

multimode non-Hermitian skin effect. I still feel obligated to say that the argument and the evidence together is not convincing, especially because the authors are trying to use the characterization of a multimode laser to support their claim on non-Hermitian skin effect through a somewhat complicated theory, and the experimental evidence is only the intensity measurements and the multi-mode spectra, with two measurement conditions for each device. For the 5-element array, the measured spectra show three unevenly spaced peaks, which is not what the theory predicts. Also, in both the 2-element array and the 5-element array, the relative intensity between the spectral peaks varied a lot when the asymmetric coupling direction was reversed. The authors did not have any explanation around this, but it makes one wonder if it is possible that the multiple modes are from detuned resonators being amplified instead of coupled modes. If the authors want to convince the readers that these are indeed skin modes in phase-locked lasers, I expect at least two more measurements. One with continuous varying of pumping region, similar to Fig. 3 and S5, but with spectra measurements. In addition, increase the pumping region to cover both upper and lower waveguides. If the theory is correct, when both upper and lower waveguides are covered, we should see an increase in the frequency splitting between coupled modes from 1nm to 6nm according to the numbers the authors used. Secondly, I would suggest the authors to pump each laser individually and show how much detuning there is between them, to exclude the possibility that the multi-mode spectrum is simply from detuning.

In addition to more experimental evidence, I think the authors need to be more transparent about how they control βL. In the article, the authors mentioned several times that they set βL to be the value of interest and they observe behavior that matches in the theory. This is very hard to believe, as the lasing wavelength itself between Fig. 3 and 4 varied by ~3 nm, showing that the fabrication quality may not be as high as the authors indicate (assuming the detuning was not intentional), also βL at these two different wavelengths would be completely different. How do the authors know which structures have βL->mπ and which ones have βL->mπ+π/2? In supplementary section 3, the authors claim "By testing samples with different link lengths, we find the sample with link length that satisfies the phase condition". But how?? The authors have not mentioned anywhere the actual lengths of the waveguides for the devices measured experimentally. I suggest that the authors make a table to list the β, L, γ_l, γ_u for all the devices that the authors presented in the manuscript, which is a critical missing link between the theory and the experiment.

Also, in this rebuttal and in the supplementary section 9, the authors claim that "Since the splitting between individual modes is less than the resolution of our spectrometer (0.64 nm), the multi-mode spectra shown in the manuscript does not resolve all the modes". However, in Fig.2, the only evidence that the array is single mode and phase-locked is the spectra. The authors cannot have both of these claims. If the spectrometer resolution is not sufficient to resolve all the modes, then it is not sufficient to prove the array is single-mode. I think both of these two claims are not convincing. Based on the authors' theory, the frequency splitting between modes can very well be below the 0.64 nm resolution when the βL is between mπ and mπ+π/2, and a multimode device can look single-mode under the spectrometer. For the multi-mode device, according to the theory authors presented in section 9, the 5 spectral peaks are approximately evenly spaced and if three of them can be resolved, I don't see where the other 2 can hide.

In conclusion, I still have to hold my recommendation of rejection, because of the severe disconnection between theory and experiment, including oversimplified claims regarding βL, the complete lack of dispersion in the model, the lack of experimental evidence regarding multimode device, and inaccurate claims regarding

spectrometer measurements.

Responses from authors -3rd Round

The concept of FSR is, for sure, not limited to resonators. One example is FSR in Mach-Zehnder interferometers. See, for example, [Chrostowski and Hochberg, Silicon photonics design: from devices to systems, Cambridge University Press, 2015] Equation (4.20). But really there is no need to debate about this, since I explicitly defined what I meant by FSR in my previous comment. Note that group index is used in Equation (4.20) of [Chrostowski and Hochberg], for the same reason that I suggested, to capture the dispersion of the waveguide. When calculating the dependence of βL on wavelength, group index should be used, instead of assuming a constant effective index which ignores the dispersion. Albeit my repeated suggestions, the authors still ignore the waveguide dispersion in their model, which results in likely a factor of ~1.7 error in their $\Delta\beta L$ vs wavelength, which is significant since the waveguide is very long, with $\beta L{\sim}266\pi$. If the authors want to claim quantitative agreement between their model and experiment, this dispersion must be considered. It actually raises one's doubt about the authors' result when they claim such high level of agreement between experiment and theory when a critical factor, waveguide dispersion, is completely ignored. The authors should use a mode solver to calculate the effective index for each wavelength (or simply, as I suggested, calculate the group index) in their structure and modify their model, if they want to claim quantitative agreement in a system with a waveguide as long as 133x wavelength. This is obviously critical for such a long waveguide and can significantly change the authors' estimation of coupling coefficients.

First, we would like to clarify that concepts like FSR cannot be arbitrarily defined for waveguides as the reviewer insinuated. If the reviewer had a good understanding about this basic concept, he/she would have noticed why it can be used in an interferometer setting as well.

As explained before and can be seen in these simulations as well, the inclusion of dispersion only causes a shift in the resonance frequency, thus the proper phase condition is met at a slightly different link length. Nevertheless, the system behaves in the same way as previously described.

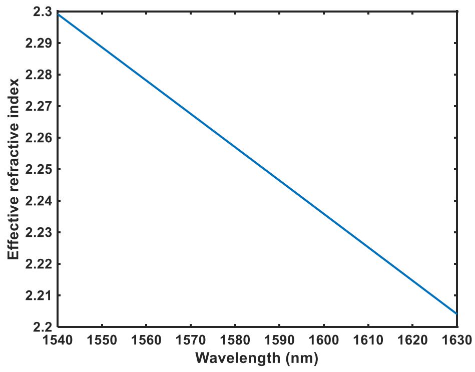

Figure FR1. Effective refractive index dispersion obtained by simulations.

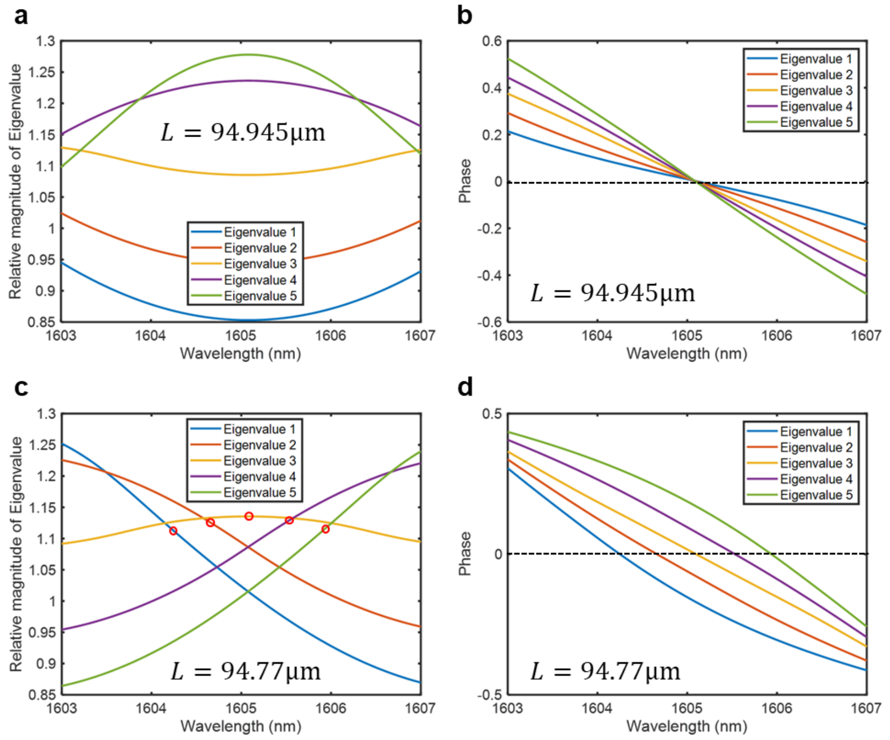

Figure FR2. Relative magnitude and phase of the eigenvalues for two different link lengths apart by $\pi/2$ phase shift with gain induced phase change. For (a) and (b), $L = 94.945\ \mu m$. For (c) and (d), $L = 94.77\ \mu m$. Red circles in (c) indicate the resonance wavelengths of individual modes.

About the detailed model that authors provided for the 5-element array, this is indeed progress towards the right direction. However, I find the argument and evidence laid out by the authors still not sufficiently convincing and far from considering publication on Light.

First of all, since the authors have repeatedly stated that my model is simple and does not accurately describe the system. Let me remind the authors that their original model, which is now still the model in the main text, is even simpler, and does not accurately describe the system either. Now that the authors accept that out of the 5 modes in Fig. RRR2 (B), only one mode satisfies βL= mπ+π/2, while two of the modes satisfy βL=mπ, it seems sufficient to say that the claims (for example around Fig. 4) about the link length satisfying βL -> mπ + π/2 is oversimplifying and confusing, if not inaccurate.

To avoid confusion, we have changed the phrase to “central wavelength meets the phase conditions”. Please see the caption of Fig.4 in the revised manuscript.

Moreover, there is still a significant disconnection between the theory and the experiment around the multimode non-Hermitian skin effect. I still feel obligated to say that the argument and the evidence together is not convincing, especially because the authors are trying to use the characterization of a multimode laser to support their claim on non-Hermitian skin effect through a somewhat complicated theory, and the experimental evidence is only the intensity measurements and the multi-mode spectra, with two measurement conditions for each device. For the 5-element array, the measured spectra show three unevenly spaced peaks, which is not what the

theory predicts. Also, in both the 2-element array and the 5-element array, the relative intensity between the spectral peaks varied a lot when the asymmetric coupling direction was reversed. The authors did not have any explanation around this, but it makes one wonder if it is possible that the multiple modes are from detuned resonators being amplified instead of coupled modes. If the authors want to convince the readers that these are indeed skin modes in phase-locked lasers, I expect at least two more measurements. One with continuous varying of pumping region, similar to Fig. 3 and S5, but with spectra measurements. In addition, increase the pumping region to cover both upper and lower waveguides. If the theory is correct, when both upper and lower waveguides are covered, we should see an increase in the frequency splitting between coupled modes from 1nm to 6nm according to the numbers the authors used. Secondly, I would suggest the authors to pump each laser individually and show how much detuning there is between them, to exclude the possibility that the multi-mode spectrum is simply from detuning. In addition to more experimental evidence, I think the authors need to be more transparent about how they control βL. In the article, the authors mentioned several times that they set βL to be the value of interest and they observe behavior that matches in the theory. This is very hard to believe, as the lasing wavelength itself between Fig. 3 and 4 varied by ~3 nm, showing that the fabrication quality may not be as high as the authors indicate (assuming the detuning was not intentional), also βL at these two different wavelengths would be completely different. How do the authors know which structures have βL->mπ and which ones have βL->mπ+π/2? In supplementary section 3, the authors claim "By testing samples with different link lengths, we find the sample with link length that satisfies the phase condition". But how?? The authors have not mentioned anywhere the actual lengths of the waveguides for the devices measured experimentally. I suggest that the authors make a table to list the β, L, γ_l, γ_u for all the devices that the authors presented in the manuscript, which is a critical missing link between the theory and the experiment.

We disagree with the Reviewer about the detuning. Not only detuning between individual lasers cannot explain the case of single mode response, it is also expected to significantly reduce the coupling efficiency and thus compromise the skin effect. Since the multi-mode results are indeed showing asymmetric coupling and skin effect in the laser array, detuning between individual lasers must be negligible.

We would like to once again emphasize that the method we used to find the proper link lengths, as indicated in the supplementary information and also pointed out by the Reviewer in his/her previous comments, is to fabricate and examine 16 samples with sweeping link lengths from $L_1 = 266\pi/\beta = 94.76\,\mu m$ to $L_{16} = 267\pi/\beta = 95.12\,\mu m$ and by doing so certainly two of the samples will meet the desired phase conditions.

Also, in this rebuttal and in the supplementary section 9, the authors claim that "Since the splitting between individual modes is less than the resolution of our spectrometer (0.64 nm), the multi-mode spectra shown in the manuscript does not resolve all the modes". However, in Fig.2, the only evidence that the array is single mode and phase-locked is the spectra. The authors cannot have both of these claims. If the spectrometer resolution is not sufficient to resolve all the modes, then it is not sufficient to prove the array is single-mode. I think both of these two claims are not convincing. Based on the authors' theory, the frequency splitting between modes can very well be below the 0.64 nm resolution when the βL is between mπ and mπ+π/2, and a multimode device can look single-mode under the spectrometer. For the multi-mode device, according to the theory authors presented in section 9, the 5 spectral peaks are approximately evenly spaced and if three of them can be resolved, I don't see where the other 2 can hide.

First, as shown in the simulation results in the supplementary information, frequency splitting in 2- and 5-

element systems are on the order of 1 nm and 0.5 nm, respectively. This is also confirmed in the experimental results in Fig. 4. Secondly, multi-mode behavior with two fairly close resonances, even though may not be resolved by the spectrometer, it can still be recognized by a broadened linewidth of the resonance (like what can be seen in Fig. 4)- which as the reviewer can easily verify is not the case in figures 2 and 3.

In conclusion, I still have to hold my recommendation of rejection, because of the severe disconnection between theory and experiment, including oversimplified claims regarding βL, the complete lack of dispersion in the model, the lack of experimental evidence regarding multimode device, and inaccurate claims regarding spectrometer measurements.

We find it surprising that a reviewer who is confused about basic concepts in optics like FSR (that by no means applies to waveguide settings) holds such a strong opinion. After all, we have repeatedly proved him/her wrong through rigorous analysis and extensive simulations for 2-element as well as 5-element systems.
